# A Precise Apple Quality Prediction Model Integrating Driving Factor Screening and BP Neural Network

**DOI:** 10.3390/plants14243795

**Published:** 2025-12-13

**Authors:** Junkai Zeng, Mingyang Yu, Yan Chen, Xin Li, Jianping Bao, Xiaoqiu Pu

**Affiliations:** 1 College of Horticulture and Forestry Science, Tarim University, Alar 843300, China; 10757251046@stumail.taru.edu.cn (J.Z.); yumingyangxj@126.com (M.Y.); 10757232053@stumail.taru.edu.cn (Y.C.); 10757251040@stumail.taru.edu.cn (X.L.); baobao-xinjiang@126.com (J.B.); 2 Southern Xinjiang Special Fruit Trees High-Quality, High-Quality Cultivation and Deep Processing of Fruit Products Processing Technical National Local Joint Engineering Laboratory, Alar 843300, China

**Keywords:** apple, quality prediction, BP neural network, feature screening, photosynthetic characteristics, sugar-acid metabolism

## Abstract

Apple fruit quality is primarily determined by Vitamin C (VC), Soluble Saccharides (SSs), Titratable Acid (TA), and the Soluble Saccharides/Titratable Acid (SSs/TA). This study aims to establish a prediction model based on the Back Propagation (BP) neural network by analyzing the intrinsic relationships between these quality indicators and the photosynthetic physiological characteristics of fruit trees, providing a new method for the precise prediction and regulation of fruit quality. Using ‘Fuji’ apple as the material, fruit quality indicators, leaf photosynthetic parameters, canopy structure indicators, and carbon–water–nitrogen metabolism indicators were systematically measured. Correlation analysis was employed to identify key influencing factors, BP neural network models with different hidden layer structures were constructed, and the optimal feature subset was screened through feature importance analysis, single-factor sensitivity analysis, and ablation experiments, ultimately establishing a simplified and efficient prediction model. Pn, Gs, SPCI, and DUE showed significant positive correlations with VC, SS, and SS/TA, whereas N and NLT were significantly positively correlated with TA content. SUE was identified as a common core driving factor for VC, SS, and SS/TA. The BP neural network demonstrated strong predictive performance for the four quality indicators, with the optimal model achieving validation set R^2^ values of 0.87, 0.86, 0.86, and 0.89, respectively. The simplified model developed through feature screening exhibited further improved performance: the validation set R^2^ for the VC prediction model increased to 0.93, while MAE and MAPE decreased by 32% and 35%, respectively. Photosynthetic characteristics and nitrogen metabolism status of the fruit trees serve as key physiological foundations determining apple quality. The quality prediction model based on the BP neural network achieved high accuracy, and its predictive performance was significantly enhanced after feature refinement, providing an effective tool for precise apple quality prediction and smart orchard management.

## 1. Introduction

Apple (*Malus domestica Borkh.*), one of the most important temperate fruits globally, has its internal fruit quality—such as vitamin C (VC) content, the composition of soluble saccharides (SSs) and titratable acid (TA), and the sugar-acid ratio (SS/TA)—directly determining the fruit’s nutritional value, flavor, and market value [1]. In recent years, with growing consumer demand for high-quality fruits, how to precisely regulate fruit quality through cultivation has become a core issue facing the apple industry. The formation of fruit quality is a complex physiological process resulting from the combined effects of genetic background, environmental factors, and cultivation management practices. Among these, photosynthesis in fruit trees and the carbon–nitrogen metabolism driven by it serve as the physiological foundation for material accumulation and the synthesis of flavor compounds [2].

Leaves are the primary organs for photosynthesis, and their photosynthetic characteristics, such as net photosynthetic rate (Pn) and stomatal conductance (Gs), directly determine the capacity for photosynthetic product formation. Concurrently, tree architecture parameters, including leaf area index (LAI), fractional interception of radiation (FIR), and the utilization efficiencies of direct and diffuse light (DUE and SUE, respectively), collectively govern the spatial distribution and utilization efficiency of light within the canopy, ultimately influencing the total amount and allocation of photosynthetic assimilates [3]. Furthermore, nitrogen, as a key constituent element of chloroplasts, enzymes, and proteins, influences not only the establishment and function of the photosynthetic apparatus but also directly participates in the anabolic pathways of quality-related components such as organic acids and amino acids. This influence is mediated through its content (N) and metabolic status, including indicators like the nitrogen limitation threshold (NLT) [4,5]. Therefore, systematically elucidating the intrinsic relationships between fruit quality and the aforementioned photosynthetic physiology and canopy structural indicators serves as the theoretical prerequisite for achieving precise quality regulation.

However, the relationships between canopy indicators and quality traits are highly nonlinear and multidimensional, forming a complex system that traditional linear statistical models (such as multiple linear regression and principal component regression) often struggle to accurately capture and interpret [6]. In recent years, artificial intelligence models have been increasingly applied in agricultural prediction, including artificial neural networks (ANN) [7], support vector machines (SVMs) [8], and Random Forest (RF) [9]. While these models demonstrate good performance under certain conditions, they still exhibit limitations when handling complex physiological and ecological data: ANN models are prone to becoming trapped in local minima and suffer from insufficient training stability [10]; SVM incurs high computational costs in high-dimensional, large-data scenarios [11]; and ensemble methods like Random Forest, although capable of handling nonlinearity, offer weak mechanistic interpretability due to their “black-box” nature [12]. These limitations hinder the practical application of existing models in precision orchard management.

In contrast, the Back Propagation (BP) neural network, which adjusts network weights via an error back-propagation algorithm, exhibits stronger nonlinear mapping capabilities and more robust learning characteristics [13,14,15]. It can autonomously extract complex features from high-dimensional data and effectively establish an end-to-end prediction model linking canopy driving factors to fruit quality, offering a novel approach to overcome the limitations of existing methods [16].

Based on this, this study proposes the following hypothesis: Integrating key canopy driving factors with the BP neural network algorithm can construct a highly accurate and strongly generalizable apple quality prediction model, which will outperform traditional linear methods and some existing AI models. To test this hypothesis, this study integrated the measurement of core apple quality indicators and key canopy physiological structure parameters, aiming to achieve the following objectives: (1) systematically analyze the intrinsic relationships between various canopy indicators and VC, SS, TA, and SS/TA; (2) construct and refine a quality prediction model based on a BP neural network, determining the optimal network architecture and training algorithm for each quality parameter; (3) identify the core driving factors influencing different quality components through feature importance analysis and ablation experiments, thereby building a streamlined and efficient refined model; and (4) validate the effectiveness and reliability of the developed models. This research aims to provide new methodological support for elucidating the formation mechanisms of apple fruit quality and for achieving accurate pre-harvest prediction, while also offering practical tools for the precision management of smart orchards.

## 2. Materials and Methods

### 2.1. Site Overview and Experimental Materials

This experiment was conducted in an apple orchard located on the campus of Tarim University in Alar, China. The orchard was oriented in an East–West row direction, with a planting spacing of 3.5 m × 1.8 m, and trees were trained to a central leader fruiting form. The test trees were approximately 3.5 m in height, with a canopy spread of 1.25–1.75 m and an operation path width of 3.5 m. The soil type in the orchard area was sandy loam soil. The study employed a field experimental design and was carried out in conjunction with statistical analysis methods.

### 2.2. Experimental Design and Sample Collection

The experiment employed a completely randomized block design. From October to November 2024, during the fruit ripening period, a total of 45 sample trees were selected for sampling. Representative fruit samples were collected uniformly from each sample tree according to four orientations (east, west, south, and north) and three canopy layers (upper, middle, and lower), yielding 225 samples in total. Concurrently, mature and healthy leaves were collected from vegetative shoots near the corresponding fruit-bearing branches for the determination of photosynthetic parameters and physiological indicators. All measurements were performed with three replicates.

### 2.3. Items and Methods of Determination

#### 2.3.1. Determination of Fruit Quality Parameters

The vitamin C content was determined using the molybdenum blue colorimetric method [17]. First, a 1 mg/mL ascorbic acid standard stock solution was prepared: 100 mg of ascorbic acid standard was accurately weighed and diluted to 100 mL with a 50 g/L oxalic acid solution. Aliquots of 0, 0.2, 0.4, 0.6, 0.8, and 1.0 mL of the stock solution were separately transferred into 10 mL centrifuge tubes, and each was adjusted to a final volume of 1.0 mL by adding 50 g/L oxalic acid solution. Subsequently, 1 mL of 5% sulfuric acid-ethanol solution and 1 mL of 5% ammonium molybdate solution were added sequentially to each tube. After thorough mixing, the reaction was carried out in a water bath at 30 °C for 30 min. The absorbance was measured at a wavelength of 705 nm, and a standard curve was plotted.

For sample analysis, 2.00 g of fruit homogenate was accurately weighed and mixed with 10 mL of pre-chilled 50 g/L oxalic acid solution. The mixture was homogenized in an ice bath and then centrifuged at 4 °C and 8000 r/min for 15 min. The resulting supernatant was collected as the test solution. A 1.0 mL aliquot of the test solution was taken, and sulfuric acid-ethanol solution and ammonium molybdate solution were added following the same procedure described above. The mixture was reacted in a water bath at 30 °C for 30 min before measurement.

The absorbance of the reaction solution was measured at a wavelength of 705 nm using a UV-Vis spectrophotometer. The vitamin C content in the sample was calculated based on the standard curve, as shown in Formula (1).

(1) $$\mathrm{Vc}=\frac{C\;\times \;V\;\times \;n}{m\;\times \;1000}\;\times \;100$$

where C is the ascorbic acid concentration (μg/mL) obtained from the standard curve; V is the total volume of the extraction solution (mL); n is the dilution factor; and m is the sample mass (g).

The soluble sugar content was determined by the anthrone colorimetric method [18]. First, a 1 mg/mL glucose standard solution was prepared by accurately weighing 100 mg of anhydrous glucose, dissolving it in distilled water, and diluting to a final volume of 100 mL. Aliquots of 0, 0.1, 0.2, 0.4, 0.6, and 0.8 mL of this solution were separately transferred into stoppered test tubes, and distilled water was added to bring the total volume in each tube to 1.0 mL. Subsequently, 4.0 mL of a freshly prepared anthrone-ethyl acetate solution (0.2 g anthrone dissolved in 100 mL ethyl acetate) and 5.0 mL of concentrated sulfuric acid were added sequentially. After thorough shaking, the mixtures were reacted in a boiling water bath for 10 min, immediately cooled in an ice bath, and the absorbance was measured at a wavelength of 630 nm to construct the standard curve.

For sample processing, 1.00 g of fruit homogenate was accurately weighed, mixed with 10 mL of an 80% ethanol solution, and extracted in an 80 °C water bath for 30 min with intermittent shaking. After cooling, the mixture was centrifuged at 4000 r/min for 10 min, and the supernatant was collected. The residue was extracted once more, and the combined supernatants were decolorized with activated carbon and then diluted to 25 mL with 80% ethanol. A 0.5 mL aliquot of the extract was taken and subjected to the color development procedure described above. The absorbance at 630 nm was measured, and the soluble sugar content was calculated according to Formula (2).

(2) $$S=\frac{C\;\times \;V\;\times \;n}{m\;\times \;10^{6}}\;\times \;100$$

The titratable acidity (TA) content was determined by neutralization titration with NaOH [19]. Precisely 5.00 g of fruit homogenate sample was weighed into a 150 mL conical flask. Then, 50 mL of freshly boiled and cooled distilled water was added, and the mixture was extracted in an 80 °C water bath for 30 min with occasional shaking. After cooling, the mixture was filtered, and the residue was washed with distilled water 3–4 times. The filtrate was collected and diluted to volume in a 100 mL volumetric flask.

Exactly 20.0 mL of the sample extract was pipetted into a 150 mL conical flask, followed by the addition of 2 drops of 1% phenolphthalein indicator. The solution was titrated with a 0.01 mol/L NaOH standard solution until a faint pink color appeared and persisted for 30 s as the endpoint. The volume of NaOH solution consumed was recorded. A blank test was performed simultaneously using distilled water.

The titratable acidity, expressed as malic acid, was calculated according to the following formula (Formula (3)).

(3) $$\mathrm{TA}=\frac{\left(V\;-\;V_{0}\right)\;\times \;C\;\times \;K\;\times {\;V}_{1}}{m\;\times \;V_{2}\;\times \;1000}$$

V is the volume of NaOH consumed in sample titration (mL); V_0_ is the volume of NaOH consumed in blank titration (mL); C is the concentration of the NaOH standard solution (mol/L); K is the conversion factor for malic acid (0.067); V_1_ is the total volume of the sample extract (mL); V_2_ is the volume of sample solution used in titration (mL); m is the sample mass (g).

Sugar-acid ratio (SS/TA): Calculated as (SS/TA) [20].

#### 2.3.2. Determination of Photosynthetic and Canopy Structural Indices in Fruit Trees

Photosynthetically active radiation (PAR) was measured using an LAI-2200C Plant Canopy Analyzer (LI-COR Biosciences, Lincoln, NE, USA) or an equivalent model [21]. The instrument was equipped with a 180° fisheye lens and calculated PAR by comparing the radiation difference above and below the canopy. Measurements were conducted under clear, cloudless weather conditions, within the time window from 2 h after sunrise to 2 h before sunset.

Prior to formal measurement, the instrument was preheated for 20 min and uniformly set to the UTC + 8 time zone to automatically record timestamps. Initial calibration was performed at an open, unobstructed area to establish a reference point, and reference PAR data above the canopy were collected. Subsequently, five measurement points were arranged beneath the canopy of each sample tree, located at the four quadrant points and the center point of the crown projection. The detector was placed horizontally at a height of 1.5 m above the ground, with the fisheye lens oriented toward the zenith. At each measurement point, three consecutive readings were recorded, and their average was taken as the PAR value for that point.

During the measurement process, radiation data were simultaneously recorded both above the canopy (in an open area) and below the canopy. The instrument automatically calculated the canopy transmittance and output the PAR value (unit: μmol·m^−2^·s^−1^). Finally, the distribution of PAR within the canopy was analyzed based on the instrument’s built-in model, with the specific calculation formula provided in Formula (4).

(4) $${PAR}_{Canopy}={PAR}_{Above}\times T_{total}$$

${PAR}_{Above}$ is the measurement value at the reference point above the canopy; $T_{total}$ is the total transmittance of the canopy (calculated by the instrument using the gap fraction model).

Leaf area index (LAI) and direct transmittance coefficient (T) were measured using an LAI-2200C plant canopy analyzer (LI-COR Biosciences, USA) [22]. The instrument is equipped with a 180° fisheye lens and a light sensor, enabling simultaneous acquisition of canopy digital images and radiation data. Measurements were conducted under clear, cloudless weather conditions between sunrise and sunset, specifically during periods when the solar elevation angle exceeded 30° and light intensity was stable (10:00–14:00), with no strong wind interference (wind speed < 3 m/s).

Prior to measurement, the instrument was preheated for 15 min, and the measurement parameters were set to 5 zenith angle rings and 4 azimuth angles. A reference point measurement was performed in an open area above the canopy: the sensor was placed horizontally at a height of 1.8 m above the ground, and three sets of PAR data were recorded consecutively. The average of these readings was taken as the reference value above the canopy (PAR_0_). For each sample tree, measurement points were arranged using the five-point method, located at the center and the east, west, south, and north directions, respectively. Each point was positioned at a distance of two-thirds of the crown radius from the main trunk. The sensor was kept horizontal with the lens facing the zenith, and three sets of valid data were acquired at each measurement point.

(5) $$LAI=\frac{ln\left(\frac{1}{T}\right)}{G\Omega }$$

(6) $$T=\frac{{\bar{PAR}}_{below}}{{PAR}_{0}}$$

In Equation (5), G is the leaf projection function (default value is 0.85); Ω is the aggregation index (default value is 0.95).

In Equation (6), T is the direct transmittance coefficient; PARbelow is the average photosynthetically active radiation below the canopy; PAR0 is the reference value above the canopy.

Net photosynthetic rate (Pn), transpiration rate (Tr), stomatal conductance (Gs), and intercellular CO_2_ concentration (Ci) were measured using an LI-6800 portable photosynthesis system (LI-COR Biosciences, USA) [23,24,25]. The instrument was equipped with a standard 2 × 3 cm transparent leaf chamber and a red-blue light source. All measurements were conducted between 9:00 and 11:00 AM under clear weather conditions.

Prior to measurement, the instrument was preheated for 30 min. The CO_2_ absorbent and desiccant were replaced, and the CO_2_/H_2_O infrared gas analyzer was calibrated. The LED light source was also checked and preheated. The measurement parameters were set as follows: photosynthetic photon flux density (PPFD) at 1000 μmol·m^−2^·s^−1^, reference chamber CO_2_ concentration maintained at 400 μmol·mol^−1^, leaf chamber temperature controlled at 25 ± 1 °C, airflow rate at 500 μmol·s^−1^, and relative humidity kept at 60 ± 5%. During measurement, current-year branches facing east within the canopy were selected. From these, fully expanded, well-illuminated, and disease-free functional leaves were chosen, and the measurement points were marked (avoiding the midrib). The leaf was flattened and clamped into the leaf chamber, ensuring complete coverage of the measurement window. After allowing parameters to stabilize (typically requiring 2–3 min), recording commenced when the rates of change for both the CO_2_ concentration difference (ΔCO_2_) and the H_2_O concentration difference (ΔH_2_O) fell below 0.5%/min. Three sets of stable data were collected consecutively at 30 S intervals, with the measured values for Pn, Tr, Gs, and Ci automatically output by the instrument.

Water use efficiency (WUE) was calculated using Equation (7) [26].

(7) $$WUE=\frac{Pn}{Tr}$$

The total nitrogen content in leaves was determined using the Kjeldahl method [27]. After drying and grinding the leaf samples, 0.2000 g was accurately weighed, mixed with a combined catalyst and concentrated sulfuric acid, and digested at 420 °C for 2 h. The digest was then distilled in a Kjeldahl distillation apparatus, absorbed with boric acid, and titrated with a 0.01 mol/L HCl standard solution. The total nitrogen content was calculated according to Formula (8).

(8) $$N=\frac{\left(V-V_{0}\right)\times C\times 0.014\times D}{m}\times 100$$

where V is the volume of hydrochloric acid consumed by the sample (mL); V0 is the volume of hydrochloric acid consumed by the blank (mL); C is the concentration of the hydrochloric acid standard solution (mol/L); D is the dilution factor (10 in this method); m is the sample mass (g); and 0.014 is the millimolar mass of nitrogen (g/mmol).

The stomatal limitation value (Ls) was calculated using Equation (9) [28].

(9) $$Ls=1-\frac{C_{i}}{C_{\alpha }}$$

$C_{i}$ is the measured intercellular CO_2_ concentration (μmol·mol^−1^); $C_{\alpha }$ is the atmospheric CO_2_ concentration, using the average measured value of 412 μmol·mol^−1^ above the canopy in this study.

Extinction coefficient (k): This parameter was calculated using the Beer-Lambert law in conjunction with LAI and the light intensity beneath the canopy, as shown in Equation (10) [29].

(10) $$k=-\frac{ln\left(\frac{I}{I_{0}}\right)}{LAI}$$

where I is the average light intensity below the canopy (μmol·m^−2^·s^−1^); I_0_ is the natural light intensity above the canopy (μmol·m^−2^·s^−1^); and LAI is the leaf area index.

Canopy fractional interception of radiation (FIR) [30], direct light use efficiency (DUE) [31], and scattered light use efficiency (SUE) [32] were calculated using Formulas (11), (12), and (13), respectively, based on measurements of photosynthetically active radiation (PAR) inside and outside the canopy.

(11) $$FIR=\left(1-\frac{{PAR}_{below}}{{PAR}_{above}}\right)\times 100\%$$

(12) $$DUE={PAR}_{above}\times FIR\times \eta_{direct}$$

(13) $$SUE={PAR}_{above}\times \left(1-FIR\right)\times \eta_{diffuse}$$

In the formula, PAR_below_ is the average photosynthetically active radiation below the canopy (μ mol·m^−2^·s^−1^); PAR_above_ is the natural photosynthetically active radiation above the canopy (μ mol·m^−2^·s^−1^); η_direct_ is the direct light conversion efficiency coefficient, with a value of 0.85; η_diffuse_ is the diffuse light conversion efficiency coefficient, with a value of 0.92.

Canopy openness (Fb) was measured using a plant canopy analyzer [33]. Multiple measurement points were evenly distributed beneath the canopy of the fruit trees. An LAI-2200C canopy analyzer (LI-COR Biosciences, Lincoln, NE, USA) was used to simultaneously record light data from a reference point above the canopy and from each measurement point below it. The instrument’s dedicated analysis software automatically calculated the canopy openness value based on the canopy porosity in the 0° zenith angle direction.

#### 2.3.3. Calculation of Carbon–Water–Nitrogen Co-Metabolism Indices

Stomatal-Photosynthetic Coupling Index (SPCI) [30,34]: Calculated based on the synergistic variation relationship between Gs and Pn, with the formula shown in Equation (14).

Nitrogen Limitation Threshold (NLT) [31,35]: Computed using a model based on the relationship between leaf nitrogen content and photosynthetic capacity, with the formula shown in Equation (15).

Carbon Cost Index (CCI) [36]: Represents the balance between carbon assimilation and consumption, with the formula shown in Equation (16).

(14) $$SPCI=\frac{G_{S}}{Pn}\times N\times 1000$$

(15) $$NLT=\frac{{Pn}_{max}-{Pn}_{actual}}{G_{S}}\times N\times 1000$$

(16) $$CCI=\frac{T_{r}}{Pn}\times N\times 1000$$

### 2.4. Construction and Validation of a BP Neural Network Model

Model structure: In terms of model construction, this study adopts a three-layer Back Propagation (BP) neural network architecture [37]. A weight and threshold iterative update mechanism is established, with the training termination conditions set to a maximum of 30 iterations or upon reaching a predefined error threshold [38]. The 18 measured physiological structure indicators (in the feature-optimized model modeling, the input indicators refer to the physiological structure indicators selected based on feature importance evaluation from ablation experiments and feature selection) serve as input variables, while individual fruit quality indicators (VC, TA, SS, and SS/TA) are used as output targets.

**Figure 1:**
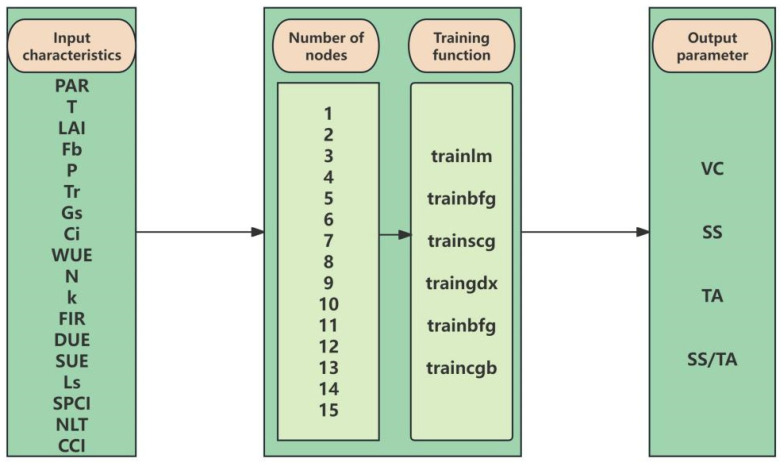
BP Neural Network Flowchart.

Regarding the choice of training function, this study encompasses six algorithms with distinct numerical characteristics: trainlm, based on Levenberg–Marquardt optimization, offers rapid convergence and is suitable for fast convergence on small to medium-sized datasets; trainbfg employs a quasi-Newton algorithm, achieving a balance between memory usage and precision; trainscg, as a scaled conjugate gradient method, is applicable for large-scale parameter optimization; traingdx combines adaptive learning rate and momentum terms, effectively avoiding local minima; traincgb enhances the stability of conjugate gradient training through the Powell–Beale restart strategy; and traincgp employs the Polak–Ribière update rule, demonstrating distinct numerical characteristics in conjugate gradient computation. The systematic comparison of these algorithms enables a comprehensive evaluation of the network’s performance under different optimization strategies [39,40]. Regarding the configuration of the number of nodes in the hidden layer, the test range was set from 5 to 15 nodes. This interval ensures the model possesses sufficient expressive capacity to capture nonlinear relationships while effectively controlling the parameter scale to prevent overfitting caused by an overly complex structure. Through this systematic parameter search, the network architecture most suitable for the complexity of this dataset can be determined [41,42], aiming to identify the optimal model structure. The specific process is illustrated in Figure 1.

In the data preparation stage, all feature data were subjected to min-max normalization (Formula (17)) and then randomly divided into a training set (158 samples) and a validation set (67 samples) at a 7:3 ratio [43].

The selection of the BP neural network as the modeling approach is primarily based on the following considerations: compared to Support Vector Regression (SVR), which faces limitations in kernel function selection for high-dimensional nonlinear systems [44], and ensemble methods such as Random Forest (RF), which exhibit shortcomings in interpretability [45], the BP network can more flexibly capture the complex nonlinear relationships between photosynthetic parameters and quality traits through its multi-layer error backpropagation mechanism. Simultaneously, when contrasted with the broader category of artificial neural networks (ANNs), the BP algorithm offers distinct advantages, including a well-defined structure, stable training processes, and ease of implementation [46], making it more suitable for the modeling requirements of the medium-sized dataset in this study. Furthermore, this algorithm has accumulated numerous successful applications in agricultural physiological prediction problems, providing methodological references for the present research.

(17) $$Q_{n}=\frac{Q-Q_{min}}{Q_{max}-Q_{min}}$$

where Q is the raw measurement data, Q_nis_ the standardized value of the raw measurement data, and Q_max_ and Q_min_ are the maximum and minimum values of the variable, respectively.

Model Training and Evaluation: The model performance was comprehensively assessed using the coefficient of determination (R^2^, Equation (18)), mean absolute error (MAE, Equation (19)), mean absolute percentage error (MAPE, Equation (20)), and model bias error (MBE, Equation (21)) [47].

(18) $$R^{2}=1-\frac{\sum_{i=1}^{n}{\left(y_{i}-\hat{y_{i}}\right)}^{2}}{\sum_{i=1}^{n}{\left(y_{i}-{\bar{y}}_{i}\right)}^{2}}$$

(19) $$MAE=\frac{1}{n}\sum_{i=1}^{n}\left|y_{i}-{\hat{y}}_{i}\right|$$

(20) $$MAPE=\frac{100\%}{n}\sum_{i=1}^{n}\left|\frac{y_{i}-{\hat{y}}_{i}}{y_{i}}\right|$$

(21) $$MBE=\frac{1}{n}\sum_{i=1}^{n}\left({\hat{y}}_{i}-y_{i}\right)$$

### 2.5. Feature Importance Analysis and Model Optimization

Feature importance ranking: The importance scores of each input feature in the trained BP network were calculated based on the connection weight method.

Single-factor sensitivity analysis and ablation experiments: By sequentially excluding individual features and re-evaluating the model performance (measured by metrics such as ΔR^2^), the sensitivity of each feature to the model prediction ability was assessed [48].

Feature selection and model simplification: Based on the feature importance scores and the results of sensitivity analysis, a core subset of features critical to the prediction target was screened. A simplified model was then reconstructed using this subset, and its performance was compared with that of the original model [49].

### 2.6. Data Analysis

All testing data were subjected to descriptive statistics and Pearson correlation analysis using Origin 2022. Neural network modeling and related computations were completed in the MATLAB R2024b environment.

## 3. Results and Analysis

### 3.1. Analysis of Data on Apple Fruit Quality and Physiological Structure Indicators

The quality indices of apple fruits, including Vc, TA, and SS contents as well as SSC/TA ratio, exhibited certain variations among different samples. Specifically, Vc content generally ranged from 0.42 to 0.90 mg/100 g, while TA and SS varied within 0.29–15.41% and 6.32–16.90%, respectively. Regarding photosynthetic characteristics, the average canopy effective radiation was approximately 516.87 μmol·m^−2^·s^−1^, and the mean leaf area index was 1.90, indicating a relatively open canopy structure overall. Among leaf photosynthetic parameters, the average net photosynthetic rate was 9.81 μmol·m^−2^·s^−1^, while the mean transpiration rate and water use efficiency were 3.72 mmol·m^−2^·s^−1^ and 2.89 μmol·mol^−1^, respectively, demonstrating favorable photosynthetic performance and moisture regulation capacity in the leaves. Parameters related to light energy utilization, such as the average light interception rate, reached 51.61%. The utilization rates of direct and scattered light were 27.70 μmol·m^−2^·s^−1^ and 0.03 μmol·m^−2^·s^−1^, respectively. Among the indicators of carbon–water–nitrogen synergistic metabolism, the average stomatal-photosynthetic coupling index was 3.27 mmol·μmol^−1^·g^−1^ (Table 1), indicating generally favorable overall metabolic coordination. Differences in fruit quality and mineral element content persisted among the orchards, which may be attributed to factors such as regional climate, tree nutritional status, and growth microenvironment.

### 3.2. Pearson Correlation Analysis

**Figure 2:**
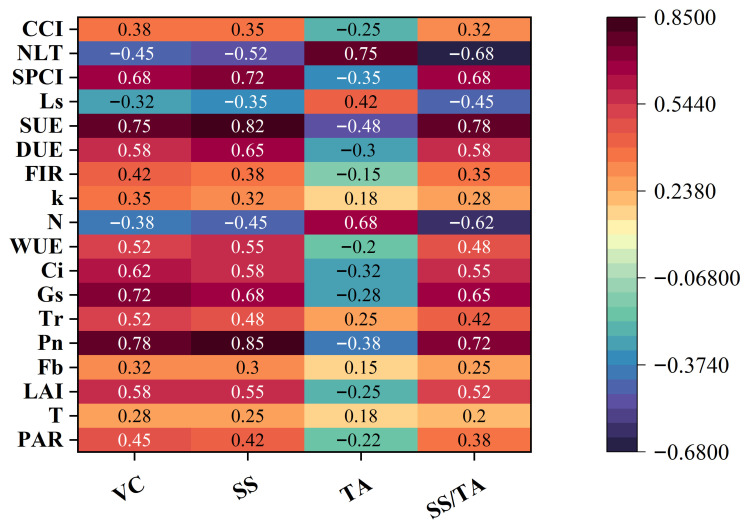
Correlation between apple fruit quality and physiological structure indicators.

Figure 2 visually illustrates the correlation patterns between 18 plant physiological and structural indicators and key fruit quality components. As “positive regulatory factors,” VC (vitamin C) and SS (soluble saccharides) enhance the net photosynthetic rate (Pn) significantly by promoting canopy development (showing a strong positive correlation with LAI) and optimizing stomatal behavior (positively correlated with Gs and SPCI), thereby improving direct light use efficiency (DUE) and water use efficiency (WUE). Specifically, the correlation coefficient between SS and Pn reaches 0.85. Ample photosynthetic products not only supply carbon skeletons and energy for VC synthesis but also directly promote the accumulation of soluble saccharides. On the other hand, because plants prioritize resource allocation for efficient direct light utilization, VC and SS exhibit a significant negative correlation with scattered light use efficiency (SUE). TA (titratable acid) acts as a “negative regulatory factor.” Its accumulation exhibits an inhibitory association with photosynthetic efficiency (negatively correlated with Pn) and canopy light interception capacity (negatively correlated with LAI and FIR). The core reason lies in the fact that the metabolic process of titratable acid competes for nitrogen resources (correlation coefficient with NLT is 0.75) and simultaneously restricts stomatal opening (positively correlated with Ls, negatively correlated with Gs), thereby indirectly reducing CO_2_ supply and diminishing photosynthetic intensity. Furthermore, the accumulation of TA likely tends to consume photosynthetic intermediates, creating a metabolic competition with sugars and VC.

SS/TA (sugar-acid ratio), as a key indicator of fruit flavor harmony, plays a dual role as both a “balancing signal” and a “final expression.” Its positive correlations with Pn, Gs, and DUE, and negative correlations with NLT and Ls, essentially reflect the synergistic interplay among photosynthetic efficiency, resource utilization, and sugar-acid metabolism. Specifically, stronger photosynthesis is associated with tighter coupling between stomatal conductance and photosynthesis, greater dominance of sugar accumulation, and a weaker inhibitory effect of titratable acid, ultimately contributing to a more favorable sugar-acid ratio.

In detail, the high correlation (0.85) between soluble saccharides (SS) and Pn indicates both the direct accumulation of soluble saccharides as photosynthetic products and their feedback regulatory role as metabolic signals on the photosynthetic process. The positive correlations of vitamin C (VC) with photosynthetic and canopy metrics confirm the dependence of its synthesis on photosynthetic products and energy. The inhibitory associations exhibited by titratable acid (TA) further clarify the specific pathway through which TA interferes with photosynthesis—by competing for nitrogen resources and imposing stomatal limitations—and ultimately affects fruit quality formation. This study provides a basis for elucidating the synthesis mechanisms of VC, soluble saccharides, and titratable acid in fruits. It also indicates that cultivation practices—such as regulating the canopy light environment and optimizing nitrogen supply—can be employed to directionally improve the fruit sugar-acid ratio, thereby enhancing fruit quality.

### 3.3. BP Neural Network Model for Predicting Vc

To predict apple Vc content based on photosynthetic data, this study systematically compared various back propagation neural network architectures. The results indicated that the model with five hidden layers and the trainlm training function demonstrated the best performance. This model achieved a coefficient of determination (R^2^) of 0.87 and a mean absolute percentage error (MAPE) of 0.0123 on the validation set, both being the optimal or near-optimal values among all models.

**Figure 3:**
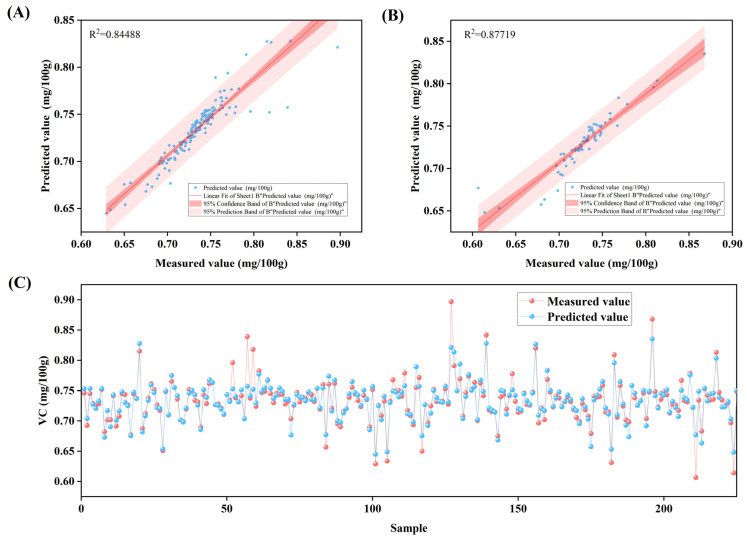
Analysis of results from the 18-5-1 structured Back Propagation (BP) neural network model with the trainlm training function: (**A**) shows the linear fitting analysis between measured values and predicted values in the Training set; (**B**) presents the linear fitting analysis between measured values and predicted values in the Validation set; (**C**) displays the visual analysis of measured values versus predicted values for Vc content across various samples.

Notably, the model exhibited comparable performance on the training set and the validation set (R^2^ values of 0.84 and 0.87, respectively), with all metrics on the validation set slightly outperforming those on the training set. This indicates good generalization ability and stability, with no signs of overfitting. In contrast, models with more hidden layers, specifically the 18-7-1 and 18-9-1 architectures, showed significant performance discrepancies between the training and validation sets, indicating clear overfitting. Although the 18-13-1 model performed stably, its prediction accuracy was limited (see Table 2 and Supplementary Table S1). The relationship between measured and predicted values shown in Figure 3 further verifies the reliability of the preferred model. Both the Training set and Validation set exhibit a high goodness of fitting, with scatter points closely distributed around the diagonal line, indicating that the model can accurately capture the variation pattern of Vc content. In summary, the trainlm model with an 18-5-1 structure, owing to its excellent generalization ability and prediction accuracy, proves to be a suitable choice for predicting apple Vc content.

### 3.4. BP Neural Network Model for Predicting Titratable Acidity

To screen for a suitable model for predicting apple titratable acid content, this study compared various neural network architectures. Overall, the model with 9 hidden layers and employing the trainbfg training function demonstrated the best comprehensive performance. This model achieved a high coefficient of determination (R^2^ = 0.86) on the validation set. More importantly, the performance difference between its training set and validation set was the smallest among all models, indicating good generalization ability and predictive stability without significant overfitting (Table 3 and Supplementary Table S2).

**Figure 4:**
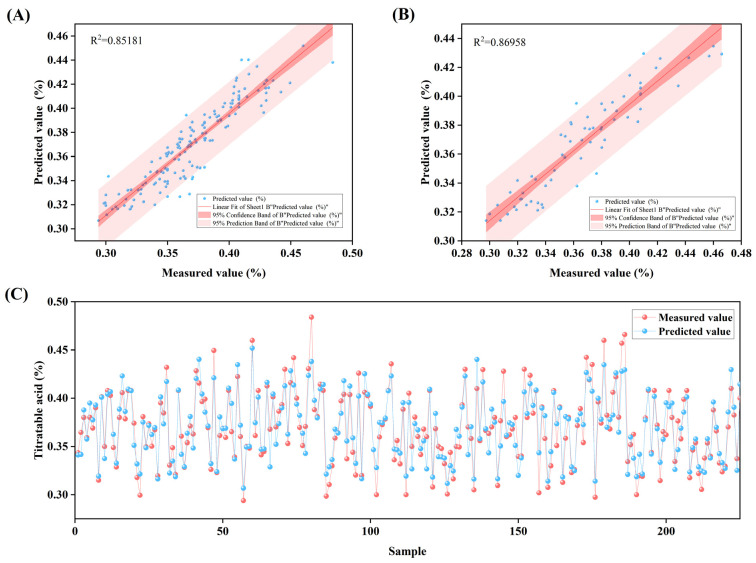
Results analysis of the 18-9-1 structured Back Propagation (BP) neural network model with trainbfg training function: (**A**) shows the linear fitting analysis between measured values and predicted values in the Training set; (**B**) presents the linear fitting analysis between measured values and predicted values in the Validation set; (**C**) displays the visual analysis of measured values versus predicted values for titratable acid content in each sample.

Based on these results, the model with an 18-9-1 architecture using the trainbfg training function was selected as the predictive tool for titratable acid content. The fitting between measured and predicted values (Figure 4A,B) shows that data points from both the training and validation sets are closely distributed along the regression line, reflecting consistent predictive performance of the model. Further examination of the sample scatter plot (Figure 4C) reveals that the predicted values for most samples are close to their measured values, providing additional support for the model’s reliable prediction accuracy in practical applications.

### 3.5. BP Neural Network Model for Predicting Soluble Saccharides

After comprehensively comparing various models, the 18-5-1 architecture paired with the trainlm training function was identified as the optimal model for predicting the soluble sugar content in apples. This model demonstrated high accuracy on the validation set (R^2^ = 0.86). Its key strength lies in the high consistency of performance metrics between the training set and the validation set, indicating excellent generalization ability and structural robustness. Compared to other candidate models, this network effectively captured the underlying patterns in the data while avoiding overfitting, thereby delivering more reliable predictions when faced with unseen data (Table 4 and Supplementary Table S3).

**Figure 5:**
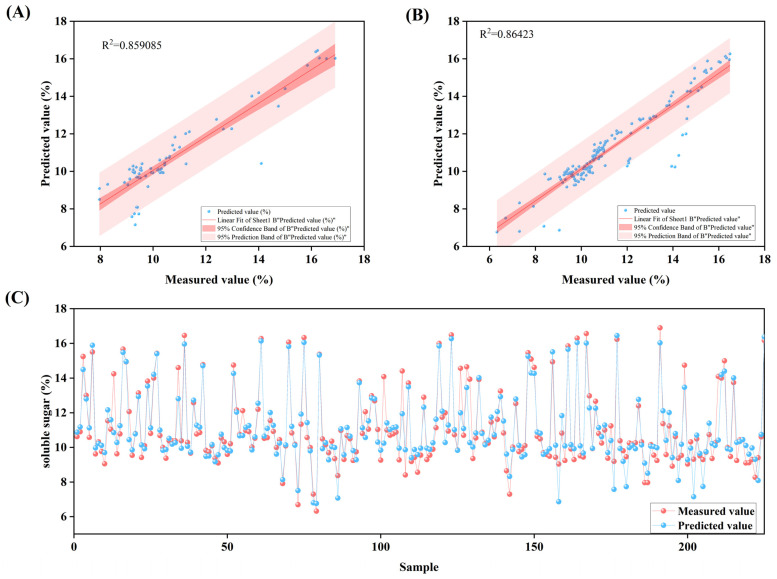
Analysis of results for the 18-5-1 structured Back Propagation (BP) neural network model with trainlm training function: (**A**) shows the linear fitting analysis between measured values and predicted values in the Training set; (**B**) presents the linear fitting analysis between measured values and predicted values in the Validation set; (**C**) displays the visual analysis of measured values versus predicted values for titratable acid content across all samples.

The fitting results between the measured values and predicted values (Figure 5A,B) further validated the model’s consistency, with data points from both the training set and validation set closely distributed around the regression line. The scatter plot of samples (Figure 5C) shows that the predicted values for most samples are close to the measured values, further corroborating the model’s accuracy and stability in practical application.

### 3.6. A BP Neural Network Model for Predicting Sugar-Acid Ratio

Following a comprehensive evaluation, the model with an 18-6-1 architecture employing the training function ‘trainbfg’ demonstrated optimal performance in predicting the sugar-acid ratio of apples. This model exhibited a high goodness of fitting (R^2^ = 0.89) during validation, with both the mean absolute error and mean relative error being the lowest among all models, indicating minimal deviation between the predicted values and the measured values. Notably, the model’s overall performance on the validation set surpassed that on the training set, suggesting it did not overfit to the specific features of the training data but instead effectively captured the general patterns underlying sugar-acid ratio variation. Consequently, it possesses more reliable generalization ability (Table 5 and Supplementary Table S4).

**Figure 6:**
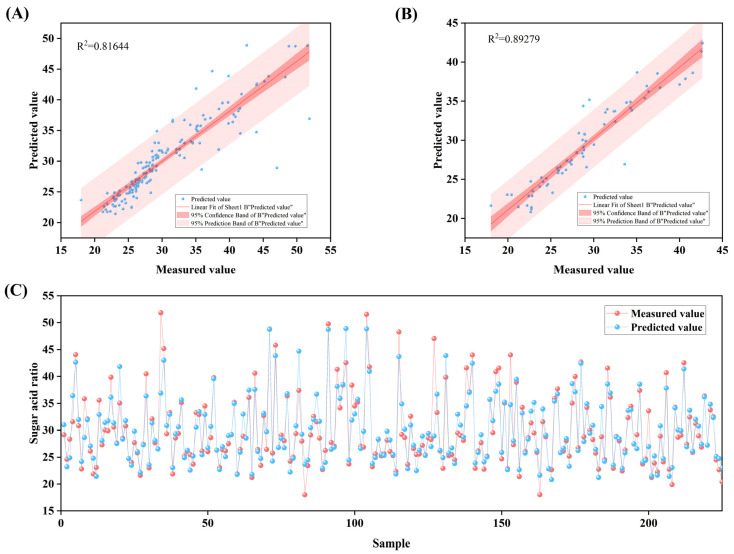
Analysis of results from the Back Propagation (BP) neural network model with 18-6-1 structure using trainbfg training function: (**A**) shows the linear fitting analysis between measured values and predicted values in the Training set; (**B**) presents the linear fitting analysis between measured values and predicted values in the Validation set; (**C**) displays the visual analysis of measured values versus predicted values for titratable acid content across samples.

The fitting between the measured values and predicted values (Figure 6A–C) shows that, for both the training set and validation set, the majority of sample points cluster closely around the regression line. This further confirms the model’s favorable accuracy and stability in predicting the sugar-acid ratio of apples.

### 3.7. Feature Importance Visualization

This study elucidates the underlying physiological mechanisms by analyzing feature importance in four prediction models for apple quality components.

**Figure 7:**
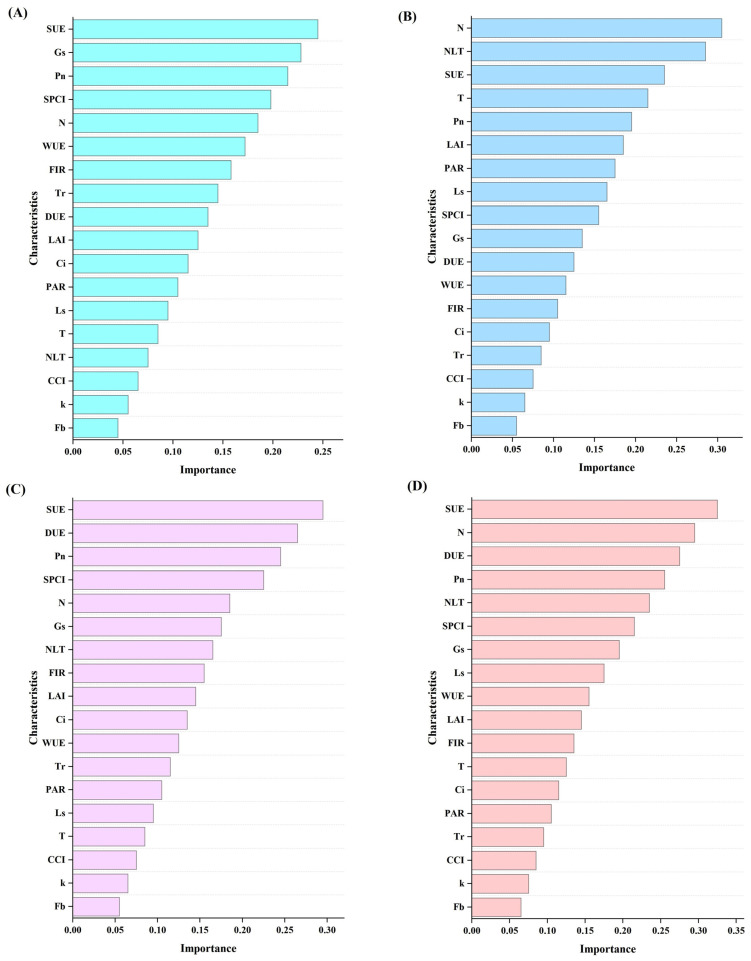
Visualization of feature importance: (**A**) shows the feature visualization for the Vc prediction model; (**B**) shows the feature visualization for the titratable acid prediction model; (**C**) shows the feature visualization for the soluble saccharides prediction model; (**D**) shows the feature visualization for the sugar-acid ratio prediction model.

In the prediction of vitamin C, the scattering light use efficiency (SUE) plays a central role, primarily by promoting the formation of precursors for VC synthesis. Photosynthesis-related parameters, such as stomatal conductance (Gs) and net photosynthetic rate (Pn), collectively influence VC accumulation by regulating carbon–nitrogen allocation and material transport processes (Figure 7A).

The titratable acid content is predominantly governed by the total leaf nitrogen content, which directly participates in acid synthesis during nitrogen metabolism. Features including the nitrogen limitation threshold (NLT) and SUE contribute to regulation by influencing enzyme activity and carbon skeleton supply (Figure 7B). In the soluble sugar model, SUE, direct light use efficiency (DUE), and Pn collectively form the core features. These three factors work synergistically within the integrated “light energy–photosynthesis–nitrogen metabolism” pathway to jointly regulate sugar accumulation (Figure 7C).

For the sugar-acid ratio prediction, SUE again emerges as the most important driving factor, influencing the sugar-acid balance by regulating the allocation of photosynthetic products. Indicators such as leaf total nitrogen and DUE represent the nitrogen and light energy utilization pathways, respectively, together constituting a coordinated regulatory network (Figure 7D).

Comprehensive analysis reveals that scattering light use efficiency (SUE) plays a central role in the formation of multiple quality traits; nitrogen-related indicators dominate titratable acid synthesis; while photosynthetic parameters serve as fundamental regulatory factors underlying the formation of all quality components.

### 3.8. One-Way Sensitivity Analysis

Based on single-factor sensitivity analysis, this study identified the most critical feature factors affecting the performance of the prediction models for each quality indicator and elucidated their underlying physiological significance.

**Figure 8:**
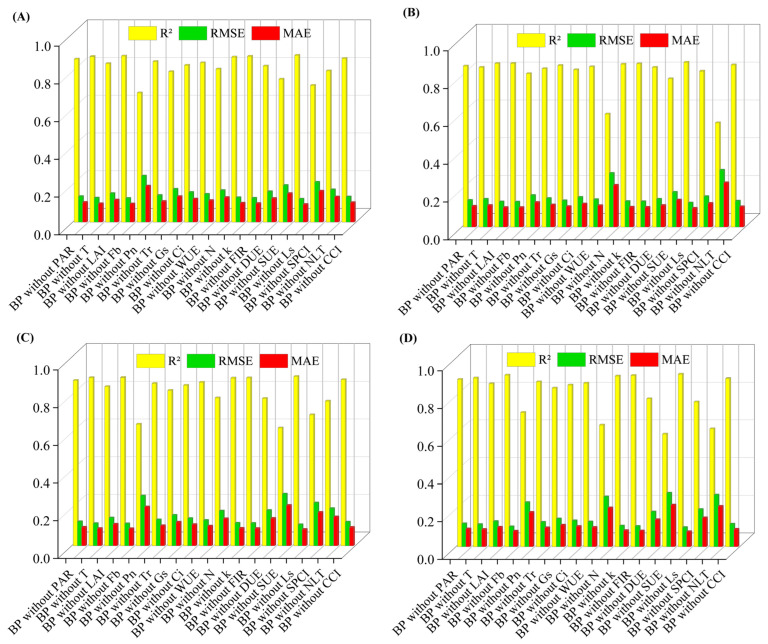
Single-factor sensitivity analysis: (**A**) shows the single-factor sensitivity analysis of the Vc prediction model; (**B**) shows the single-factor sensitivity analysis of the titratable acid prediction model; (**C**) shows the single-factor sensitivity analysis of soluble saccharides; (**D**) shows the single-factor sensitivity analysis of the sugar-acid ratio prediction model.

In the vitamin C prediction model (Figure 8A), net photosynthetic rate (Pn), stomatal-photosynthesis coupling index (SPCI), and scattered light utilization efficiency (SUE) exhibited high sensitivity. These three factors correspond, respectively, to carbon skeleton supply, photosynthetic coordination efficiency, and light energy utilization capacity. Their absence leads to a significant decline in model accuracy, indicating their irreplaceable driving roles in VC synthesis. Moderately sensitive factors such as stomatal conductance (Gs) and leaf nitrogen content are primarily involved in regulating substance transport and metabolic balance; their removal has a relatively limited impact on the model. In contrast, low-sensitivity factors such as the stomatal limitation value (Ls) reflect background environmental information; their removal has minimal impact on model performance and may even slightly improve the fitting effect in specific cases by reducing model complexity.

The titratable acid model shows high dependence on nitrogen status (Figure 8B). High-sensitivity factors, including the nitrogen limitation threshold (NLT) and leaf total nitrogen content (N), are directly linked to acid synthesis and metabolism. Medium-sensitivity factors such as SUE and Pn play auxiliary regulatory roles by influencing carbon allocation and the supply of photosynthetic products.

In the models for soluble saccharides and the sugar-acid ratio (Figure 8C,D), parameters related to photosynthesis and light-use efficiency—such as SUE, Pn, and SPCI—generally exhibit high sensitivity, further confirming the central role of photosynthetic assimilation in sugar accumulation and sugar-acid balance. Meanwhile, nitrogen-related parameters also show relatively high sensitivity in the sugar-acid ratio model, indicating that sugar-acid composition is jointly regulated by the coordinated interaction of carbon and nitrogen metabolism. Overall, the sensitivity structure of different models aligns closely with their corresponding physiological mechanisms. Vitamin C synthesis relies more heavily on photosynthetic carbon fixation and light energy conversion; titratable acid content is primarily regulated by nitrogen metabolism; while the sugar-acid balance depends on the synergistic interaction between light energy utilization and nitrogen supply. These findings provide a clear rationale for model simplification: when constructing practical prediction models, medium- and high-sensitivity features can be prioritized, effectively reducing data collection and computational costs while maintaining high accuracy.

### 3.9. Feature Importance Assessment and Feature Selection Based on Ablation Experiments

Based on single-factor sensitivity analysis and ablation experiments, this study identified the optimal feature subsets for the prediction models of each quality indicator.

**Figure 9:**
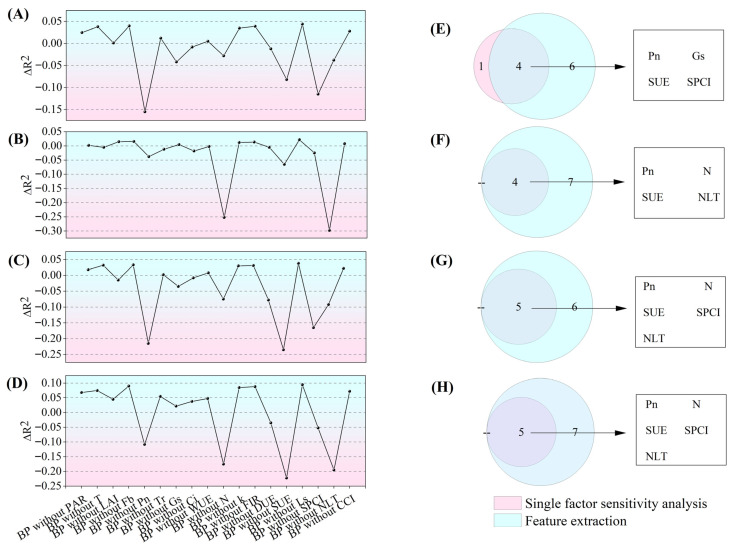
Feature importance assessment and feature selection based on ablation experiments: (**A**) shows the single-factor sensitivity analysis and ablation experiment results for the Vc prediction model; (**B**) shows the single-factor sensitivity analysis and ablation experiment results for the titratable acid prediction model; (**C**) shows the single-factor sensitivity analysis and ablation experiment results for the soluble saccharides prediction model; (**D**) shows the single-factor sensitivity analysis and ablation experiment results for the sugar-acid ratio prediction model; (**E**) presents the intersection analysis between the single-factor sensitivity analysis results and the features used in modeling for the Vc prediction model; (**F**) presents the intersection analysis between the single-factor sensitivity analysis results and the features used in modeling for the titratable acid prediction model; (**G**) presents the intersection analysis between the single-factor sensitivity analysis results and the features used in modeling for the soluble saccharides prediction model; (**H**) presents the intersection analysis between the single-factor sensitivity analysis results and the features used in modeling for the sugar-acid ratio prediction model.

For the Vc prediction model (Figure 9A), five key features—Pn, Gs, SUE, SPCI, and NLT—were selected. This combination retains all core driving factors (Pn, SPCI, SUE) while incorporating Gs and NLT to enhance informational completeness. Redundant features were removed, improving model robustness without compromising predictive performance.

The core features of the titratable acid model focus on nitrogen metabolism-related indicators (Figure 9B), with the final subset consisting of NLT, N, SUE, and Pn. Among these, NLT and N were prioritized as primary regulators of acid synthesis, whereas SUE and Pn were included as auxiliary indicators reflecting light energy utilization and carbon supply. The optimal subset for the soluble saccharides prediction model comprised Pn, SUE, SPCI, DUE, and NLT (Figure 9C). These features collectively encompassed multiple key pathways, including photosynthesis, light energy utilization, and nitrogen regulation, thereby forming a systematic representation of sugar synthesis.

For the sugar-acid ratio model, the selected feature collection consisted of SUE, NLT, N, Pn, and SPCI (Figure 9D). This combination simultaneously reflected the integrated effects of light energy allocation and nitrogen regulation on the sugar-acid balance.

By further intersecting the results of sensitivity analysis with the modeling features, the core drivers for each quality indicator were definitively identified: Vc was primarily regulated by Pn, Gs, SUE, and SPCI (Figure 9E); the key influencing factors for titratable acid were Pn, N, SUE, and NLT (Figure 9F); and soluble saccharides and the sugar-acid ratio were both systematically regulated by Pn, N, SUE, SPCI, and NLT (Figure 9G,H). These findings provide a basis for feature selection in constructing concise and efficient prediction models for apple quality.

### 3.10. Feature Verification

To validate the effectiveness of feature screening, this study reconstructed prediction models based on the key feature set obtained from the screening. As shown in Table 6, the refined models demonstrated superior performance in predicting all quality indicators.

In the vitamin C prediction task, the new model maintained its accuracy on the training set while significantly enhancing its generalization ability. The goodness of fitting on the validation set increased to 0.93, and error metrics decreased by over 30%, indicating that removing redundant variables helps the model focus more on key physiological processes.

For the prediction of titratable acid, the new model achieved performance improvements on both the training and validation sets, with errors significantly reduced. Moreover, the prediction bias remained close to zero, suggesting that the model enhanced explanatory power without introducing systematic error. In the prediction of soluble saccharides, the refined model also demonstrated outstanding performance, with the goodness of fitting for both the Training set and the Validation set exceeding 0.90. The validation error was reduced by approximately one-quarter, reflecting the model’s enhanced Generalization ability and stability.

The improvement in sugar-acid ratio prediction was particularly notable. While maintaining a high goodness of fitting, the new model effectively corrected the systematic bias present in the original model, resulting in more accurate and reliable predictions.

Overall, the simplified model constructed through feature screening not only has a more compact structure but also outperforms the original model in terms of generalization ability, error control, and systematic bias correction. This validates the effectiveness and practical value of the feature screening strategy employed in this study.

## 4. Discussion

### 4.1. Photosynthetic Physiology and Carbon–Nitrogen Metabolism Basis of Apple Fruit Quality Formation

This study systematically measured the quality indices of apple fruits and the related physiological and structural parameters of the trees. Correlation analysis revealed that photosynthetic characteristics, such as net photosynthetic rate (Pn) and stomatal conductance (Gs), showed significant positive correlations with vitamin C (VC) content, soluble saccharides (SS) content, and the sugar-acid ratio (SS/TA), but a negative correlation with titratable acid (TA) content [50]. This finding underscores that photosynthesis serves as the material and energetic foundation for fruit quality formation. Specifically, a higher Pn directly promotes the accumulation of photosynthetic products, providing sufficient carbon skeletons and ATP for the synthesis of VC and SS [51]. Concurrently, the positive correlation between Gs and SPCI indicates that refined stomatal behavior ensures effective CO_2_ supply and coordinates with the photosynthetic process, thereby further enhancing this mechanism [52].

Notably, the effects of different light resource utilization modes on quality components vary. Direct light use efficiency (DUE) shows a positive correlation with soluble sugar (SS) content and the sugar-acid ratio, whereas scattered light use efficiency (SUE) exhibits a strong negative correlation with vitamin C (VC) and SS content. This discrepancy may stem from the resource allocation strategy formed by plants under the regulation of canopy structure: in environments with sufficient direct light, photosynthetic products tend to be efficiently converted into substances such as sugars; in environments dominated by scattered light or with a closed canopy, although the SUE value increases, the overall light energy capture and conversion efficiency is limited, potentially leading to a relative insufficiency of resources allocated for the synthesis of VC and SS [53].

On the other hand, nitrogen metabolism plays a central role in regulating fruit acidity. Leaf total nitrogen content (N) and nitrogen limitation threshold (NLT) showed a strong positive correlation with TA content, but a negative correlation with the sugar-acid ratio. This confirms that nitrogen serves as a crucial precursor and driving force for titratable acid synthesis. When nitrogen supply is sufficient, fruit trees preferentially allocate photosynthetic products and nitrogen resources to the synthesis of organic acids (such as malic acid). This process may compete with sugar synthesis for substrates, thereby explaining the negative correlations observed between TA and SS or VC. Such synergy and trade-off between carbon and nitrogen metabolism ultimately determine the sugar-acid balance and flavor quality of the fruit [54].

### 4.2. Applicability and Refinement of BP Neural Network Model in Fruit Quality Prediction

This study successfully developed BP neural network models for predicting VC, TA, SS, and SS/TA, respectively. Each optimal model demonstrated excellent performance on the validation set (R^2^ > 0.86), confirming the feasibility of reliably predicting fruit internal quality based on photosynthetic physiology data. Compared with traditional linear regression models, the BP neural network was more effective in capturing the complex nonlinear relationships between various physiological indicators and quality components [55]. Notably, the BP models in this study exhibited considerable competitiveness when compared to other intelligent algorithms applied in recent agricultural research. For instance, in the study by Song et al. (2025), Random Forest models typically reported an R^2^ of 0.73, while Support Vector Regression models achieved an R^2^ of 0.65 [56]. The accuracy level achieved by this model indicates that BP neural networks possess unique advantages in handling such complex agricultural biological data.

Through feature importance analysis and single-factor sensitivity analysis, we found that the core driving factors for different quality components vary, which aligns with the aforementioned physiological mechanisms. VC synthesis is highly dependent on SUE, Pn, and SPCI, highlighting the central role of light resource utilization efficiency and photosynthetic synergy [57]; TA accumulation is primarily driven by N and NLT, underscoring the critical function of nitrogen metabolism; SS and SS/TA are jointly regulated by photosynthetic factors such as SUE and Pn, as well as nitrogen-related factors including N and NLT, reflecting the integrated influence of carbon–nitrogen co-metabolism [58].

Based on the aforementioned analysis, feature screening significantly enhanced model efficiency and simplicity. The optimal feature subset identified through ablation experiments, after removing redundant and noisy variables, yielded a refined model that not only exhibited a more streamlined structure but also demonstrated superior prediction accuracy and generalization ability compared to the original model utilizing all features. Taking VC prediction as an example, the coefficient of determination (R^2^) on the validation set for the refined model increased from 0.87 to 0.93, while the mean absolute error (MAE) decreased by 32%. These results demonstrate that feature screening grounded in physiological mechanisms enables the model to focus more effectively on core patterns, effectively mitigating overfitting and consequently exhibiting greater robustness when confronted with unseen data [59].

### 4.3. Model Limitations

The prediction model developed in this study demonstrates potential for application in apple quality assessment, yet several limitations warrant further refinement. The model was trained on sampling data from 45 trees and 225 fruits, a sample size and diversity that remain insufficient. This foundational dataset may inadequately capture the comprehensive effects of different apple stocks, growing regions, and interannual climatic variations on fruit quality formation. Consequently, the model requires more extensive external validation before practical deployment [60].

Regarding variable interpretation, although input parameters were optimized through feature screening, the complex intrinsic relationships between photosynthetic indices and quality traits warrant cautious consideration. Taking the scattering light use efficiency (SUE) as an example, its dominant role observed in multiple models may stem from its specific physiological function in light energy utilization under low-light conditions. Alternatively, it could be attributed to coupling effects with unquantified factors such as canopy structure and foliage distribution [61]. This potential multicollinearity issue suggests that when applying the model for production decision-making, it is necessary to further discern the true contribution of variables through field experiments.

It should be noted that the current model has not yet incorporated key environmental factors such as soil physicochemical properties, moisture dynamics, and microclimate data. These elements exert direct regulatory effects on photosynthetic metabolism and substance conversion, and their absence may limit the model’s explanatory power in complex cultivation environments.

### 4.4. Implications for Cultivation Management and Research Prospects

The findings of this study provide clear guidance for precision cultivation management in orchards. First, by monitoring key photosynthetic parameters such as Pn, Gs, and SUE, early prediction and assessment of fruit VC and sugar content can be achieved [62]. Second, to improve fruit flavor (by increasing the sugar-to-acid ratio), cultivation practices should focus on both “promoting sugar” and “controlling acid”: “promoting sugar” requires improving canopy structure through proper pruning to enhance DUE and Pn; “controlling acid” necessitates precise regulation of nitrogen fertilizer application to avoid excessive nitrogen leading to over-accumulation of organic acids [63].

Future research could be further developed in the following aspects: First, environmental factors such as soil nutrients and moisture could be incorporated into the model to build a more comprehensive quality prediction system. Second, the model established in this study is based on a specific dataset, and its generalizability requires further validation and calibration across different stocks, regions, and climatic conditions. Third, exploring the integration of such prediction models with intelligent orchard management systems could provide decision-making support for targeted regulation of fruit quality and intelligent production.

## 5. Conclusions

The apple quality prediction model based on photosynthetic physiological parameters developed in this study offers a novel technical pathway for precision management in smart orchards. By measuring key indicators such as net photosynthetic rate and scattered light utilization efficiency, and integrating an optimized BP neural network, the model enables effective prediction of intrinsic fruit quality attributes—including vitamin C content and sugar-acid composition—prior to harvest.

At the practical application level, this model can directly support orchard management decisions. By monitoring core parameters such as Pn and SUE, growers can promptly assess the physiological status of trees, providing a basis for precise fertilization and pruning. For instance, when the model predicts a low sugar-acid ratio, nitrogen fertilizer application can be adjusted to improve the allocation of photosynthetic products. Meanwhile, abnormal fluctuations in SUE can serve as a reference signal for optimizing canopy structure. It is important to acknowledge that the current model still has certain limitations. First, the training data for the model were all sourced from a single production region and have not been validated under different ecological conditions. Its generalization ability requires further confirmation through cross-regional external testing. Second, the model has not yet integrated environmental data such as soil moisture and meteorological factors. The mechanisms by which these factors influence quality formation need to be quantified in subsequent research.

Future research efforts will focus on advancing three key directions: (1) conducting joint trials across multiple stock varieties and production regions to establish a quality prediction standard with broad applicability; (2) exploring the integration of unmanned aerial vehicle (UAV) remote sensing and Internet of Things (IoT) sensor data into the model to extend predictions from the individual tree to the orchard scale; and (3) developing an intelligent decision-making system that integrates quality prediction and cultivation regulation, ultimately forming a scalable smart orchard solution. In summary, the method proposed in this study lays a foundation for the targeted regulation of fruit quality. However, transforming it into a mature application technology still requires continuous advancement in areas such as model generalizability, data integration, and system integration.

## Figures and Tables

**Table 1:** Data on fruit quality indicators and physiological structure indicators used in the artificial neural network model.

Parameter Metrics	Maximum	Minimum	Average	SD	CV
Vc (mg/100 g)	0.90	0.42	0.73	0.04	16.75
TA (%)	15.41	0.29	0.87	2.23	0.39
SS (%)	16.90	6.32	11.15	2.21	5.04
SS/TA	51.85	0.70	28.98	9.92	2.92
PAR (μmol·m^−2^·s^−1^)	1135.00	100.50	516.87	243.79	2.12
T	0.93	0.06	0.48	0.21	2.35
LAI (Leaf Area Index)	5.13	0.28	1.90	1.05	1.82
Fb	1.03	0.06	0.59	0.18	3.37
Pn (μmol·m^−2^·s^−1^)	15.20	6.43	9.81	1.74	5.65
Tr (mmol·m^−2^·s^−1^)	6.23	0.71	3.72	0.74	5.05
Gs (mmol·m^−2^·s^−1^)	322.13	106.20	226.77	47.09	4.82
Ci (μmol·mol^−1^)	379.30	252.84	308.56	19.70	15.66
WUE (μmol·mol^−1^)	21.47	1.65	2.89	1.78	1.62
N (%)	2.85	1.67	2.22	0.17	13.08
k	1.54	0.06	0.48	0.23	2.06
FIR (%)	94.40	7.30	51.61	20.59	2.51
DUE (μmol·m^−2^·s^−1^)	164.41	8.83	27.70	24.31	1.14
SUE (μmol·m^−2^·s^−1^)	0.12	0.01	0.03	0.02	1.39
Ls	0.37	0.05	0.23	0.05	4.64
SPCI (mmol·μmol^−1^·g^−1^)	6.47	1.28	3.27	0.96	3.39
NLT (μmol·mmol^−1^·g^−1^)	880.03	70.67	130.61	77.91	1.68
CCI (mmol·μmol^−1^·g^−1^)	1.74	0.07	0.67	0.24	2.77

**Table 2:** Best Model Metrics for Different Numbers of Hidden Layers in VC.

Model	Training Function	Training Set				Validation Set			
		R^2^	MAE	MBE	MAPE	R^2^	MAE	MBE	MAPE
18-5-1	trainlm	0.84	0.0075	0.0002	0.0100	0.87	0.0087	0.0014	0.0123
18-6-1	trainbfg	0.83	0.0082	0.0005	0.0112	0.83	0.0106	0.0027	0.0142
18-7-1	trainlm	0.89	0.0073	0.0007	0.0099	0.83	0.0119	0.0017	0.0164
18-8-1	trainlm	0.84	0.0104	0.0038	0.0143	0.86	0.0096	0.0045	0.0131
18-9-1	trainlm	0.87	0.0064	0.0028	0.0087	0.76	0.0097	0.0039	0.0126
18-10-1	traincgb	0.85	0.0098	0.0002	0.0132	0.74	0.0120	0.0006	0.0166
18-11-1	trainlm	0.83	0.0088	0.0041	0.0121	0.84	0.0102	0.0049	0.0138
18-12-1	trainlm	0.82	0.0098	0.0014	0.0133	0.70	0.0144	0.0042	0.0195
18-13-1	trainlm	0.82	0.0087	0.0016	0.0118	0.81	0.0105	0.0052	0.0142
18-14-1	trainbfg	0.79	0.0113	0.0008	0.0156	0.73	0.0112	0.0017	0.0147

**Table 3:** Best Model Metrics for Titratable Acid at Different Numbers of Hidden Layers.

Model	Training Function	Training Set				Validation Set			
		R^2^	MAE	MBE	MAPE	R^2^	MAE	MBE	MAPE
18-5-1	traingdx	0.80	0.0143	0.0001	0.0391	0.73	0.0166	0.0005	0.0445
18-10-1	trainlm	0.86	0.0108	0.0005	0.0295	0.81	0.0130	0.0045	0.0336
18-7-1	traincgb	0.88	0.0102	0.0001	0.0277	0.78	0.0119	0.0021	0.0318
18-8-1	trainlm	0.86	0.0113	0.0043	0.0298	0.82	0.0110	0.0008	0.0301
18-9-1	trainbfg	0.85	0.0110	0.0004	0.0299	0.86	0.0118	0.0010	0.0320
18-10-1	trainscg	0.85	0.0117	0.0001	0.0315	0.79	0.0150	0.0010	0.0407
18-11-1	trainbfg	0.84	0.0116	0.0003	0.0317	0.79	0.0144	0.0041	0.0368
18-12-1	trainlm	0.86	0.0106	0.0017	0.0290	0.86	0.0128	0.0045	0.0340
18-13-1	trainlm	0.86	0.0119	0.0014	0.0317	0.79	0.0141	0.0001	0.0388
18-14-1	trainlm	0.83	0.0121	0.0083	0.0331	0.71	0.0151	0.0017	0.0422
18-15-1	trainlm	0.85	0.0118	0.0007	0.0319	0.82	0.0136	0.0011	0.0365

**Table 4:** Best model metrics for soluble saccharides under different hidden layer numbers.

Model	Training Function	Training Set				Validation Set			
		R^2^	MAE	MBE	MAPE	R^2^	MAE	MBE	MAPE
18-5-1	trainlm	0.86	0.5041	0.0490	0.0455	0.86	0.6180	0.0307	0.0597
18-3-1	traincgb	0.84	0.6501	0.0506	0.0583	0.85	0.6567	0.1337	0.0610
18-7-1	trainscg	0.88	0.5703	0.0072	0.0502	0.73	0.7587	0.0762	0.0681
18-8-1	trainlm	0.87	0.6131	0.0372	0.0559	0.71	0.8351	0.1362	0.0761
18-9-1	trainlm	0.85	0.6143	0.1011	0.0542	0.78	0.7443	0.2234	0.0632
18-10-1	trainlm	0.83	0.6629	0.0319	0.0589	0.83	0.7506	0.0607	0.0716
18-11-1	trainlm	0.83	0.6207	0.0573	0.0549	0.80	0.7201	0.0846	0.0636
18-12-1	trainlm	0.83	0.5323	0.0517	0.0472	0.77	0.7708	0.0462	0.0669
18-13-1	trainlm	0.85	0.5509	0.0484	0.0481	0.80	0.7438	0.1191	0.0654
18-14-1	trainlm	0.82	0.6766	0.0173	0.0595	0.80	0.6529	0.3181	0.0652
18-15-1	trainlm	0.87	0.5256	0.1129	0.0476	0.83	0.6307	0.0102	0.0572

**Table 5:** Best model metrics for sugar-acid ratio under different numbers of hidden layers.

Model	Training Function	Training Set				Validation Set			
		R^2^	MAE	MBE	MAPE	R^2^	MAE	MBE	MAPE
18-5-1	trainbfg	0.82	1.8773	0.0208	0.0594	0.85	1.5828	0.5326	0.0530
18-6-1	trainbfg	0.82	1.7794	0.0604	0.0549	0.89	1.3047	0.3220	0.0462
18-7-1	trainlm	0.85	1.6657	0.2146	0.0541	0.74	2.2626	0.3976	0.0727
18-8-1	trainbfg	0.80	1.6131	0.0484	0.0501	0.85	1.8743	0.0669	0.0617
18-9-1	trainscg	0.84	1.8162	0.1742	0.0584	0.77	1.9337	0.0528	0.0607
18-10-1	trainlm	0.79	1.9817	0.0451	0.0633	0.84	2.2791	0.4635	0.0731
18-11-1	traincgb	0.84	1.6363	0.0160	0.0495	0.85	1.5334	0.2238	0.0554
18-12-1	trainscg	0.84	1.6081	0.0043	0.0520	0.81	1.7187	0.0343	0.0533
18-13-1	traincgb	0.81	1.7639	0.0330	0.0557	0.88	1.7814	0.4217	0.0586
18-14-1	trainlm	0.84	1.5890	0.1955	0.0511	0.80	1.9845	0.5522	0.0625
18-15-1	trainlm	0.82	1.8166	0.6325	0.0565	0.79	1.8908	0.1562	0.0630

**Table 6:** Comparison between the feature refinement model and the original model.

Parameter Metrics	Model Configuration	Dataset	R^2^	MAE	MBE	MAPE
Vc	4-Feature Optimization Model	Training Set	0.85	0.007	0.0003	0.0095
		Validation Set	0.93	0.0059	0.0002	0.008
	8-5-1 Original Model	Training Set	0.84	0.0075	0.0002	0.01
		Validation Set	0.87	0.0087	0.0014	0.0123
TA	4-Feature Optimization Model	Training Set	0.90	0.0085	0.0002	0.022
		Validation Set	0.91	0.009	0.0003	0.024
	18-9-1 Original Model	Training Set	0.85	0.011	0.0004	0.0299
		Validation Set	0.86	0.0118	0.001	0.032
SS	5-Feature Optimization Model	Training Set	0.91	0.421	0.0201	0.037
		Validation Set	0.90	0.4625	0.015	0.048
	18-5-1 original model	Training Set	0.86	0.5041	0.049	0.0455
		Validation Set	0.86	0.618	0.0307	0.0597
SS/TA	5-Feature Optimization Model	Training Set	0.88	1.35	0.035	0.045
		Validation Set	0.90	0.90	0.018	0.035
	18-6-1 Original Model	Training Set	0.82	1.7794	0.0604	0.0549
		Validation Set	0.89	1.3047	0.322	0.0462

## Data Availability

The data that support the findings of this study are available from the corresponding author upon reasonable request.

## Supplementary Materials

The following supporting information can be downloaded at: https://www.mdpi.com/article/10.3390/plants14243795/s1, Table S1: Model metric indicators of Vitamin C under different hidden layer numbers; Table S2: Model metric indicators of Titratable Acid under different hidden layer numbers; Table S3: Model metric indicators of soluble sugars under different hidden layer numbers; Table S4: Model metric indicators of. Saccharides/Titratable Acid under different hidden layer numbers.

## Abbreviations

The following abbreviations are used in this manuscript:

VC	Vitamin C
SS	Soluble Saccharides
SS/TA	Soluble Saccharides/Titratable Acid
TA	Titratable Acid
BP	Back Propagation
PAR	Photosynthetically Active Radiation
T	Temperature
Pn	Net Photosynthetic Rate
Tr	Transpiration Rate
Gs	Stomatal Conductance
Ci	Intercellular CO_2_ Concentration
WUE	Water Use Efficiency
Ls	Stomatal Limitation Value
LAI	Leaf Area Index
Fb	Canopy Openness
k	Extinction Coefficient
FIR	Fraction of Intercepted Radiation
DUE	Direct Light Use Efficiency
SUE	Scattered Light Use Efficiency
N	Nitrogen Content
SPCI	Specific Leaf Nitrogen Content
NLT	Nitrogen Limitation Threshold
CCI	Chlorophyll Content Index
ANN	Artificial Neural Network
RF	Random Forest
SVM	Support Vector Machine
