# Supplementary material for: A Precise Apple Quality Prediction Model Integrating Driving Factor Screening and BP Neural Network
Source: Plants (Basel). 2025 Dec 13;14(24):3795. doi: 10.3390/plants14243795 (PMC12737044; doi:10.3390/plants14243795)
Supplement: Supplementary file 1 [file plants-14-03795-s001.zip › plants-4000328-supplementary.pdf]

Supplementary Materials

Table S1. Model metric indicators of Vitamin C under different hidden layer numbers.

Number of Hidden Layer Neurons:5	Training Set					Validation Set			
	R2	MAE	MBE	MAPE		R2	MAE	MBE	MAPE
Levenberg-Marquardt (trainlm)	0.8439	0.0075	-0.0002	1.00%		0.8705	0.0087	0.0014	1.23%
Traingd	0.1858	0.0217	0.0016	2.96%		0.2689	0.0234	0.0043	3.24%
Trainscg	0.5787	0.0154	0.0007	2.09%		0.5293	0.0164	0.0053	2.32%
Traingdx	0.0142	0.0259	-0.0025	3.51%		0.2209	0.0247	0.0024	3.48%
Trainbfg	0.8094	0.0092	-0.001	1.22%		0.8586	0.0089	0	1.25%
Traincgb	0.7789	0.0112	-0.0007	1.52%		0.7593	0.012	0.0015	1.70%

Number of Hidden Layer Neurons:6	Training Set					Validation Set			
	R2	MAE	MBE	MAPE		R2	MAE	MBE	MAPE
Levenberg-Marquardt (trainlm)	0.842	0.0068	-0.0013	0.94%		0.7721	0.0116	-0.0005	1.54%
Traingd	invalid value	invalid value	invalid value	invalid value		invalid value	invalid value	invalid value	invalid value
Trainscg	0.6045	0.0142	-0.0002	1.94%		0.6418	0.0164	-0.0027	2.22%
Traingdx	0.7854	0.0094	-0.0005	1.29%		0.7863	0.0127	0.0012	1.74%
Trainbfg	0.8303	0.0082	-0.0005	1.12%		0.8331	0.0106	-0.0027	1.42%
Traincgb	0.7486	0.0104	0.0001	1.43%		0.7396	0.0131	0.0013	1.79%

Number of Hidden Layer Neurons:7	Training Set					Validation Set			
	R2	MAE	MBE	MAPE		R2	MAE	MBE	MAPE
Levenberg-Marquardt (trainlm)	0.8877	0.0073	-0.0007	0.99%		0.8329	0.0119	0.0017	1.64%
Traingd	0.1098	0.0214	0.0004	2.93%		0.3412	0.0252	-0.0034	3.47%
Trainscg	0.7022	0.0118	-0.0007	1.60%		0.6722	0.0159	0.0029	2.21%
Traingdx	0.7187	0.013	0.0007	1.77%		0.7387	0.0148	0.0036	2.06%
Trainbfg	0.7753	0.0101	-0.0016	1.36%		0.7937	0.0116	0.002	1.60%
Traincgb	0.7831	0.011	0.0009	1.49%		0.7364	0.0126	0.0004	1.72%

Number of Hidden Layer Neurons:8	Training Set					Validation Set			
	R2	MAE	MBE	MAPE		R2	MAE	MBE	MAPE
Levenberg-Marquardt (trainlm)	0.8372	0.0104	0.0038	1.43%		0.855	0.0096	0.0045	1.31%
Traingd	invalid value	invalid value	invalid value	invalid value		invalid value	invalid value	invalid value	invalid value
Trainscg	0.7394	0.0138	-0.0013	1.87%		0.6625	0.0142	-0.0014	1.94%
Traingdx	invalid value	invalid value	invalid value	invalid value		invalid value	invalid value	invalid value	invalid value
Trainbfg	0.6142	0.0149	-0.0017	2.03%		0.7064	0.0125	-0.0035	1.72%
Traincgb	0.7501	0.0121	-0.0016	1.64%		0.8133	0.0115	-0.0013	1.59%

Number of Hidden Layer Neurons:9	Training Set					Validation Set			
	R2	MAE	MBE	MAPE		R2	MAE	MBE	MAPE
Levenberg-Marquardt (trainlm)	0.8672	0.0064	-0.0028	0.87%		0.762	0.0097	-0.0039	1.26%
Traingd	0.085	0.0234	0.0002	3.18%		0.1779	0.0253	-0.0029	3.43%
Trainscg	0.6407	0.0126	-0.0005	1.74%		0.6125	0.017	0.0011	2.37%
Traingdx	0.6549	0.0123	-0.0006	1.69%		0.6384	0.0146	0.0025	2.03%
Trainbfg	0.7352	0.012	0.0001	1.65%		0.6892	0.0149	-0.0005	2.06%
Traincgb	0.7809	0.0099	-0.0001	1.36%		0.7099	0.0132	0.0002	1.81%

Number of Hidden Layer Neurons:10	Training Set					Validation Set			
	R2	MAE	MBE	MAPE		R2	MAE	MBE	MAPE
Levenberg-Marquardt (trainlm)	0.8998	0.0072	-0.0003	0.97%		0.6382	0.0131	0.0021	1.78%
Traingd	invalid value	invalid value	invalid value	invalid value		invalid value	invalid value	invalid value	invalid value
Trainscg	0.6784	0.0132	-0.0001	1.81%		0.5977	0.0138	-0.0004	1.94%
Traingdx	0.6888	0.0133	-0.0007	1.80%		0.4567	0.0166	0.0026	2.30%
Trainbfg	0.8055	0.0102	-0.0004	1.38%		0.7277	0.0124	-0.0017	1.72%
Traincgb	0.8471	0.0098	0.0002	1.32%		0.7447	0.012	0.0006	1.66%

Number of Hidden Layer Neurons:11	Training Set					Validation Set			
	R2	MAE	MBE	MAPE		R2	MAE	MBE	MAPE
Levenberg-Marquardt (trainlm)	0.833	0.0088	-0.0041	1.21%		0.8405	0.0102	-0.0049	1.38%
Traingd	0.1146	0.0232	-0.0003	3.17%		0.3574	0.0254	0.0031	3.48%
Trainscg	0.7897	0.0102	0.0003	1.39%		0.7312	0.0139	-0.0014	1.92%

	Training Set					Validation Set			
Traingdx	invalid value	invalid value	invalid value	invalid value		invalid value	invalid value	invalid value	invalid value
Trainbfg	0.7568	0.0109	0.0011	1.48%		0.7736	0.0124	0.0005	1.70%
Traincgb	0.7815	0.0092	0.0006	1.26%		0.7742	0.012	0.0031	1.66%

Number of Hidden Layer Neurons:12	Training Set					Validation Set			
	R2	MAE	MBE	MAPE		R2	MAE	MBE	MAPE
Levenberg-Marquardt (trainlm)	0.8184	0.0098	-0.0014	1.33%		0.701	0.0144	-0.0042	1.95%
Traingd	invalid value	invalid value	invalid value	invalid value		invalid value	invalid value	invalid value	invalid value
Trainscg	0.6616	0.0137	0.0009	1.85%		0.6734	0.0154	0.003	2.10%
Traingdx	0.7185	0.0124	-0.0019	1.70%		0.6788	0.0147	-0.0012	2.04%
Trainbfg	0.7723	0.0109	0.0007	1.50%		0.627	0.0149	0.0057	2.05%
Traincgb	0.7209	0.0118	0.0005	1.59%		0.6197	0.0168	0.0063	2.26%

Number of Hidden Layer Neurons:13	Training Set					Validation Set			
	R2	MAE	MBE	MAPE		R2	MAE	MBE	MAPE
Levenberg-Marquardt (trainlm)	0.8188	0.0087	0.0016	1.18%		0.8078	0.0105	0.0052	1.42%
Traingd	invalid value	invalid value	invalid value	invalid value		invalid value	invalid value	invalid value	invalid value
Trainscg	0.6829	0.0142	-0.0017	1.93%		0.5595	0.017	-0.0001	2.30%
Traingdx	0.684	0.0129	-0.0017	1.77%		0.7572	0.0126	-0.0033	1.72%
Trainbfg	0.5106	0.0189	-0.0029	2.59%		0.5071	0.0182	-0.0004	2.47%
Traincgb	0.5474	0.0185	-0.0048	2.51%		0.5696	0.0176	-0.0038	2.39%

Number of Hidden Layer Neurons:14	Training Set					Validation Set			
	R2	MAE	MBE	MAPE		R2	MAE	MBE	MAPE
Levenberg-Marquardt (trainlm)	0.8361	0.0101	0.0001	1.42%		0.6895	0.0118	-0.0014	1.55%
Traingd	invalid value	invalid value	invalid value	invalid value		invalid value	invalid value	invalid value	invalid value
Trainscg	0.8225	0.0104	-0.0008	1.43%		0.634	0.0131	-0.0048	1.72%
Traingdx	0.7299	0.0137	0	1.90%		0.6799	0.0125	-0.0039	1.65%
Trainbfg	0.7855	0.0113	-0.0008	1.56%		0.7301	0.0112	-0.0017	1.47%
Traincgb	0.8629	0.0096	-0.0008	1.33%		0.6146	0.0143	-0.0015	1.89%

Number of Hidden Layer Neurons:15	Training Set					Validation Set			
	R2	MAE	MBE	MAPE		R2	MAE	MBE	MAPE
Levenberg-Marquardt (trainlm)	0.8746	0.0089	0.0027	1.20%		0.7877	0.0113	0.0025	1.55%
Traingd	invalid value	invalid value	invalid value	invalid value		invalid value	invalid value	invalid value	invalid value
Trainscg	0.7011	0.0144	-0.0012	1.98%		0.542	0.0167	-0.0058	2.28%
Traingdx	invalid value	invalid value	invalid value	invalid value		invalid value	invalid value	invalid value	invalid value
Trainbfg	0.597	0.0176	0.0017	2.38%		0.5077	0.0177	0.004	2.38%
Traincgb	0.8313	0.0096	0.0008	1.32%		0.7737	0.0107	0.0002	1.43%

Table S2. Model metric indicators of Titratable Acid under different hidden layer numbers.

Number of Hidden Layer Neurons:5	Training Set					Validation Set			
	R2	MAE	MBE	MAPE		R2	MAE	MBE	MAPE
Levenberg-Marquardt (trainlm)	0.7761	0.0154	0.0096	4.24%		0.7352	0.0157	0.0093	4.32%
Traingd	0.5413	0.0204	-0.0006	5.48%		0.4025	0.0219	-0.0033	5.72%
Trainscg	0.4846	0.0215	-0.0103	5.61%		0.3157	0.0244	-0.0135	6.32%
Traingdx	0.8025	0.0143	-0.0001	3.91%		0.7343	0.0166	-0.0005	4.45%
Trainbfg	0.5563	0.0204	-0.0026	5.48%		0.5459	0.0201	-0.0041	5.29%
Traincgb	0.769	0.0152	-0.0011	4.09%		0.6509	0.0185	-0.0013	4.92%

Number of Hidden Layer Neurons:6	Training Set					Validation Set			
	R2	MAE	MBE	MAPE		R2	MAE	MBE	MAPE
Levenberg-Marquardt (trainlm)	0.8592	0.0108	0.0005	2.95%		0.8091	0.013	-0.0045	3.36%
Traingd	invalid value	invalid value	invalid value	invalid value		invalid value	invalid value	invalid value	invalid value
Trainscg	0.7248	0.0159	0.0012	4.33%		0.6709	0.0181	-0.0051	4.72%
Traingdx	0.7548	0.0146	-0.0037	3.95%		0.6685	0.0183	-0.0101	4.80%
Trainbfg	0.7705	0.0142	-0.0008	3.86%		0.7089	0.0171	-0.0072	4.48%
Traincgb	0.7272	0.0151	-0.004	4.05%		0.5947	0.0203	-0.0109	5.21%

Number of Hidden Layer Neurons:7	Training Set					Validation Set			
	R2	MAE	MBE	MAPE		R2	MAE	MBE	MAPE
Levenberg-Marquardt (trainlm)	0.8515	0.0119	-0.0013	3.18%		0.7492	0.0131	-0.0028	3.47%
Traingd	invalid value	invalid value	invalid value	invalid value		invalid value	invalid value	invalid value	invalid value
Trainscg	0.5359	0.0214	-0.0072	5.74%		0.4766	0.0203	-0.0064	5.37%

Traingdx	0.6039	0.0193	-0.0005	5.14%		0.5244	0.0181	-0.0035	4.82%
Trainbfg	0.4771	0.0224	0.0024	6.05%		0.2349	0.024	0.0028	6.49%
Traincgb	0.8815	0.0102	-0.0001	2.77%		0.7804	0.0119	-0.0021	3.18%

Number of Hidden Layer Neurons:8	Training Set					Validation Set			
	R2	MAE	MBE	MAPE		R2	MAE	MBE	MAPE
Levenberg-Marquardt (trainlm)	0.8626	0.0113	-0.0043	2.98%		0.8228	0.011	-0.0008	3.01%
Traingd	0.5509	0.0211	0.003	5.65%		0.3674	0.0218	0.008	6.03%
Trainscg	0.7511	0.0162	0.0003	4.32%		0.6919	0.0151	0.0078	4.16%
Traingdx	0.8077	0.0137	-0.0011	3.69%		0.6691	0.0154	0.0044	4.31%
Trainbfg	0.7749	0.0154	0.001	4.16%		0.683	0.0156	0.0078	4.36%
Traincgb	0.8446	0.0127	0.0011	3.42%		0.8035	0.0123	0.0065	3.42%

Number of Hidden Layer Neurons:9	Training Set					Validation Set			
	R2	MAE	MBE	MAPE		R2	MAE	MBE	MAPE
Levenberg-Marquardt (trainlm)	0.8629	0.009	0.0023	2.40%		0.7352	0.0129	0.0043	3.45%
Traingd	0.3081	0.0251	-0.0003	6.78%		0.4318	0.025	0.0048	6.84%
Trainscg	0.6972	0.0163	-0.0012	4.36%		0.7414	0.016	0.0002	4.29%
Traingdx	0.798	0.013	0.0007	3.50%		0.7627	0.0156	0.0022	4.22%
Trainbfg	0.8517	0.011	0.0004	2.99%		0.864	0.0118	0.001	3.20%
Traincgb	0.7812	0.0136	-0.0033	3.66%		0.7823	0.0151	-0.0021	4.06%

Number of Hidden Layer Neurons:10	Training Set					Validation Set			
	R2	MAE	MBE	MAPE		R2	MAE	MBE	MAPE
Levenberg-Marquardt (trainlm)	0.8416	0.009	-0.0007	2.45%		0.7339	0.0133	-0.0019	3.70%
Traingd	0.32	0.0243	-0.0002	6.62%		0.2478	0.0256	0.0005	7.06%
Trainscg	0.8454	0.0117	0.0001	3.15%		0.7929	0.015	0.001	4.07%
Traingdx	invalid value	invalid value	invalid value	invalid value		invalid value	invalid value	invalid value	invalid value
Trainbfg	0.6235	0.0192	0.0002	5.21%		0.5815	0.021	0.0015	5.79%
Traincgb	0.7488	0.0154	-0.0013	4.13%		0.7443	0.0167	-0.0003	4.53%

Number of Hidden Layer Neurons:11	Training Set					Validation Set			
	R2	MAE	MBE	MAPE		R2	MAE	MBE	MAPE
Levenberg-Marquardt (trainlm)	0.7908	0.0125	-0.0032	3.38%		0.6708	0.0178	-0.0082	4.50%
Traingd	0.4442	0.0205	-0.0029	5.53%		0.3843	0.025	-0.0074	6.40%
Trainscg	0.785	0.013	-0.002	3.54%		0.743	0.0155	-0.0051	3.92%
Traingdx	0.6153	0.0179	0.0017	4.87%		0.45	0.0241	-0.0052	6.17%
Trainbfg	0.8359	0.0116	-0.0003	3.17%		0.7913	0.0144	-0.0041	3.68%
Traincgb	0.6635	0.0171	-0.0002	4.65%		0.5519	0.022	-0.0014	5.69%

Number of Hidden Layer Neurons:12	Training Set					Validation Set			
	R2	MAE	MBE	MAPE		R2	MAE	MBE	MAPE
Levenberg-Marquardt (trainlm)	0.8641	0.0106	0.0017	2.90%		0.8564	0.0128	0.0045	3.40%
Traingd	0.5221	0.0204	0.001	5.65%		0.6315	0.0194	0.0035	5.10%
Trainscg	0.7394	0.0153	-0.0001	4.16%		0.7124	0.0185	0.0021	4.92%
Traingdx	invalid value	invalid value	invalid value	invalid value		invalid value	invalid value	invalid value	invalid value
Trainbfg	0.7261	0.0158	0.0012	4.28%		0.6519	0.0205	0.0023	5.45%
Traincgb	0.7851	0.0137	-0.0006	3.70%		0.7563	0.0165	0.003	4.38%

Number of Hidden Layer Neurons:13	Training Set					Validation Set			
	R2	MAE	MBE	MAPE		R2	MAE	MBE	MAPE
Levenberg-Marquardt (trainlm)	0.8586	0.0119	-0.0014	3.17%		0.7855	0.0141	0.0001	3.88%
Traingd	0.4138	0.0234	0.0015	6.36%		0.3414	0.0231	0.002	6.33%
Trainscg	0.8524	0.0113	-0.0007	3.03%		0.7245	0.0148	0.0008	4.04%
Traingdx	invalid value	invalid value	invalid value	invalid value		invalid value	invalid value	invalid value	invalid value
Trainbfg	0.7633	0.0157	0.0014	4.26%		0.6374	0.0184	0.0022	5.10%
Traincgb	0.7986	0.0139	-0.0005	3.78%		0.639	0.0176	0.001	4.84%

Number of Hidden Layer Neurons:14	Training Set					Validation Set			
	R2	MAE	MBE	MAPE		R2	MAE	MBE	MAPE
Levenberg-Marquardt (trainlm)	0.8333	0.0121	0.0083	3.31%		0.7107	0.0151	0.0017	4.22%
Traingd	0.5455	0.0189	-0.0006	5.17%		0.5413	0.0241	-0.0071	6.53%
Trainscg	0.7978	0.0126	-0.0011	3.40%		0.7632	0.0176	-0.0063	4.69%
Traingdx	0.7582	0.0138	-0.0006	3.73%		0.6929	0.0197	-0.0037	5.31%

Trainbfg	0.7347	0.0147	-0.0005	4.00%	0.7042	0.0189	-0.0031	4.98%
Traincgb	0.7412	0.0147	0.0002	4.01%	0.7042	0.0189	-0.0042	5.04%

Number of Hidden Layer Neurons:15	Training Set				Validation Set			
	R2	MAE	MBE	MAPE	R2	MAE	MBE	MAPE
Levenberg-Marquardt (trainlm)	0.8468	0.0118	0.0007	3.19%	0.8154	0.0136	0.0011	3.65%
Traingd	0.4279	0.0237	0.0002	6.46%	0.2702	0.0243	0.0019	6.50%
Trainscg	0.6887	0.0173	0.0009	4.70%	0.6902	0.0178	0.003	4.77%
Traingdx	0.5376	0.0204	0.0011	5.53%	0.4112	0.0231	0.0036	6.17%
Trainbfg	0.7231	0.0149	-0.0011	3.98%	0.6947	0.0169	-0.0014	4.46%
Traincgb	0.8105	0.0133	-0.0005	3.56%	0.8236	0.0138	-0.002	3.65%

Table S3. Model metric indicators of soluble sugars under different hidden layer numbers.

Number of Hidden Layer Neurons:5	Training Set				Validation Set			
	R2	MAE	MBE	MAPE	R2	MAE	MBE	MAPE
Levenberg-Marquardt (trainlm)	0.8635	0.5041	-0.049	4.55%	0.8574	0.618	-0.0307	5.97%
Traingd	0.0445	1.68	0.1371	15.17%	0.067	1.8195	0.5057	16.67%
Trainscg	0.7387	0.901	0.0257	8.26%	0.8396	0.7109	0.0114	6.45%
Traingdx	invalid value	invalid value	invalid value	invalid value	invalid value	invalid value	invalid value	invalid value
Trainbfg	0.2759	1.4959	0.1942	13.07%	0.2552	1.6047	0.2079	14.36%
Traincgb	0.0775	1.6437	-0.1057	14.41%	0.0904	1.7603	0.273	15.62%

Number of Hidden Layer Neurons:6	Training Set				Validation Set			
	R2	MAE	MBE	MAPE	R2	MAE	MBE	MAPE
Levenberg-Marquardt (trainlm)	0.8294	0.6792	0.1328	6.14%	0.8163	0.7156	0.1424	6.69%
Traingd	0.2827	1.6089	0.049	14.13%	0.2562	1.6765	0.2279	15.38%
Trainscg	0.8358	0.6489	-0.015	5.86%	0.8382	0.6712	-0.0064	6.36%
Traingdx	0.294	1.6033	-0.1073	14.20%	0.3685	1.5265	0.2478	13.82%
Trainbfg	0.8194	0.6884	0.002	6.20%	0.8298	0.6918	0.0191	6.48%
Traincgb	0.8401	0.6501	0.0506	5.83%	0.8463	0.6567	0.1337	6.10%

Number of Hidden Layer Neurons:7	Training Set				Validation Set			
	R2	MAE	MBE	MAPE	R2	MAE	MBE	MAPE
Levenberg-Marquardt (trainlm)	0.8601	0.5687	-0.1851	4.92%	0.69	0.7133	-0.1676	6.45%
Traingd	0.3893	1.3159	-0.1042	11.05%	0.3526	1.3415	0.1153	12.00%
Trainscg	0.8826	0.5703	-0.0072	5.02%	0.7305	0.7587	-0.0762	6.81%
Traingdx	0.8644	0.636	-0.0647	5.71%	0.7266	0.7946	-0.1182	7.32%
Trainbfg	0.8711	0.6036	0.0487	5.41%	0.7473	0.7639	0.036	6.90%
Traincgb	0.8712	0.6196	0.0453	5.65%	0.6964	0.8239	0.048	7.78%

Number of Hidden Layer Neurons:8	Training Set				Validation Set			
	R2	MAE	MBE	MAPE	R2	MAE	MBE	MAPE
Levenberg-Marquardt (trainlm)	0.8654	0.6131	0.0372	5.59%	0.7118	0.8351	0.1362	7.61%
Traingd	0.1706	1.6752	-0.0685	14.89%	0.1352	1.6344	0.3082	14.85%
Trainscg	0.3676	1.4629	-0.0381	12.70%	0.346	1.3826	0.0845	11.86%
Traingdx	0.8576	0.6602	0.0098	6.09%	0.7237	0.8748	0.0803	8.01%
Trainbfg	0.851	0.6463	0.0063	5.91%	0.7044	0.8411	0.0959	7.87%
Traincgb	0.8448	0.6607	-0.0659	6.07%	0.7412	0.8103	0.0178	7.31%

Number of Hidden Layer Neurons:9	Training Set				Validation Set			
	R2	MAE	MBE	MAPE	R2	MAE	MBE	MAPE
Levenberg-Marquardt (trainlm)	0.8505	0.6143	-0.1011	5.42%	0.7768	0.7443	-0.2234	6.32%
Traingd	0.1841	1.5899	-0.0858	14.34%	0.1058	1.8458	-0.0953	16.40%
Trainscg	0.6275	1.0567	0.0246	9.36%	0.401	1.3151	0.1952	11.43%
Traingdx	0.3319	1.4091	-0.0863	12.49%	0.114	1.7221	-0.0366	14.99%
Trainbfg	0.4483	1.3159	0.1117	11.69%	0.2949	1.5704	0.1744	13.65%
Traincgb	0.8396	0.6304	-0.0477	5.85%	0.7965	0.6895	-0.2176	5.93%

Number of Hidden Layer Neurons:10	Training Set				Validation Set			
	R2	MAE	MBE	MAPE	R2	MAE	MBE	MAPE
Levenberg-Marquardt (trainlm)	0.8255	0.6629	0.0319	5.89%	0.8318	0.7506	0.0607	7.16%
Traingd	0.0002	1.5739	-0.0203	13.49%	0.0599	1.5986	0.0029	14.61%
Trainscg	0.8203	0.6299	0.0338	5.63%	0.8423	0.6596	0.0265	6.17%
Traingdx	invalid value	invalid value	invalid value	invalid value	invalid value	invalid value	invalid value	invalid value

Trainbfg	0.5497	1.2126	-0.0031	10.70%		0.602	1.2703	-0.0654	11.29%
Traincgb	0.8208	0.6603	-0.0699	5.96%		0.8176	0.7797	-0.0154	7.39%

Number of Hidden Layer Neurons:11	Training Set					Validation Set			
	R2	MAE	MBE	MAPE		R2	MAE	MBE	MAPE
Levenberg-Marquardt (trainlm)	0.8318	0.6207	-0.0573	5.49%		0.7993	0.7201	-0.0846	6.36%
Traingd	0.5254	1.3256	-0.098	12.00%		0.459	1.3357	-0.1398	11.82%
Trainscg	0.7053	1.0314	0.0162	9.28%		0.6244	1.1582	0.0619	10.44%
Traingdx	0.7534	0.8407	0.0627	7.68%		0.7245	0.8727	0.0838	7.94%
Trainbfg	0.7822	0.8282	0.1228	7.55%		0.8417	0.7193	0.1818	6.48%
Traincgb	0.7945	0.7679	-0.028	6.84%		0.7825	0.8493	0.1005	7.65%

Number of Hidden Layer Neurons:12	Training Set					Validation Set			
	R2	MAE	MBE	MAPE		R2	MAE	MBE	MAPE
Levenberg-Marquardt (trainlm)	0.8287	0.5323	0.0517	4.72%		0.7719	0.7708	0.0462	6.69%
Traingd	0.525	1.123	-0.0479	10.25%		0.5737	1.2795	-0.384	11.34%
Trainscg	0.3258	1.3118	-0.0132	11.38%		0.2733	1.5745	-0.4062	13.05%
Traingdx	0.7033	0.9184	-0.0883	8.25%		0.7109	1.134	-0.3091	9.85%
Trainbfg	0.7436	0.8617	0.0984	7.80%		0.6969	1.0752	-0.0235	9.43%
Traincgb	0.6459	0.9804	0.0232	9.03%		0.5675	1.2621	-0.2163	10.89%

Number of Hidden Layer Neurons:13	Training Set					Validation Set			
	R2	MAE	MBE	MAPE		R2	MAE	MBE	MAPE
Levenberg-Marquardt (trainlm)	0.8479	0.5509	-0.0484	4.81%		0.8033	0.7438	-0.1191	6.54%
Traingd	invalid value	invalid value	invalid value	invalid value		invalid value	invalid value	invalid value	invalid value
Trainscg	0.6166	1.0875	-0.121	9.75%		0.6054	1.2378	-0.1694	11.13%
Traingdx	0.7293	0.87	-0.0614	7.95%		0.6193	1.0935	-0.194	9.61%
Trainbfg	0.756	0.8683	0.1478	8.03%		0.6293	1.09	0.1857	9.98%
Traincgb	0.7257	0.9161	0.0146	8.35%		0.6003	1.1797	0.0128	10.95%

Number of Hidden Layer Neurons:14	Training Set					Validation Set			
	R2	MAE	MBE	MAPE		R2	MAE	MBE	MAPE
Levenberg-Marquardt (trainlm)	0.8233	0.6766	0.0173	5.95%		0.8039	0.6529	0.3181	6.52%
Traingd	0.3768	1.3982	-0.077	12.46%		0.356	1.2596	0.4885	12.61%
Trainscg	0.6941	1.0122	-0.0481	8.93%		0.5734	1.0191	0.4212	10.23%
Traingdx	0.8161	0.7	-0.0137	6.17%		0.7694	0.6949	0.3177	7.28%
Trainbfg	0.3802	1.5534	0.1093	13.63%		0.3192	1.4142	0.7126	13.78%
Traincgb	0.6705	1.0737	0.0922	9.56%		0.6176	0.9928	0.4873	9.81%

Number of Hidden Layer Neurons:15	Training Set					Validation Set			
	R2	MAE	MBE	MAPE		R2	MAE	MBE	MAPE
Levenberg-Marquardt (trainlm)	0.8692	0.5256	-0.1129	4.76%		0.8323	0.6307	-0.0102	5.72%
Traingd	0.2666	1.553	-0.0136	13.98%		0.2288	1.493	0.0997	13.81%
Trainscg	0.3598	1.38	-0.1896	12.14%		0.2509	1.5156	-0.2364	13.43%
Traingdx	invalid value	invalid value	invalid value	invalid value		invalid value	invalid value	invalid value	invalid value
Trainbfg	0.8115	0.7517	0.0479	6.84%		0.7792	0.8096	0.1503	7.21%
Traincgb	0.8272	0.6615	0.0669	6.12%		0.7417	0.7959	0.2188	7.42%

Table S4. Model metric indicators of .Saccharides /Titratable Acid under different hidden layer numbers.

Number of Hidden Layer Neurons:5	Training Set					Validation Set			
	R2	MAE	MBE	MAPE		R2	MAE	MBE	MAPE
Levenberg-Marquardt (trainlm)	0.818	1.9165	-0.3638	5.97%		0.8412	1.633	-0.0572	5.25%
Traingd	0.2022	4.8773	0.084	16.17%		0.1578	4.1054	0.6237	13.86%
Trainscg	0.7631	2.3463	0.2464	7.53%		0.7434	2.0645	1.1067	7.03%
Traingdx	0.6618	3.1573	0.1067	10.29%		0.6035	3.0795	1.0381	10.64%
Trainbfg	0.8232	1.8773	-0.0208	5.94%		0.8536	1.5828	0.5326	5.30%
Traincgb	0.7694	2.5164	0.1378	8.22%		0.7528	2.2555	0.3525	7.76%

Number of Hidden Layer Neurons:6	Training Set					Validation Set			
	R2	MAE	MBE	MAPE		R2	MAE	MBE	MAPE
Levenberg-Marquardt (trainlm)	0.8184	2.0187	0.702	6.60%		0.7792	2.0504	1.3406	7.67%
Traingd	0.284	4.6227	-0.0333	14.97%		0.3116	3.9284	0.7229	13.70%
Trainscg	0.7571	2.523	-0.1452	8.03%		0.8175	2.0441	0.0822	7.12%
Traingdx	0.1492	5.185	0.8643	17.27%		0.0638	4.4726	1.8772	16.88%

Trainbfg	0.8164	1.7794	-0.0604	5.49%		0.8892	1.3047	0.322	4.62%
Traincgb	0.8179	1.8719	0.1344	5.89%		0.8572	1.64	0.7272	5.87%

Number of Hidden Layer Neurons:7	Training Set					Validation Set			
	R2	MAE	MBE	MAPE		R2	MAE	MBE	MAPE
Levenberg-Marquardt (trainlm)	0.8549	1.6657	0.2146	5.41%		0.7375	2.2626	-0.3976	7.27%
Traingd	invalid value	invalid value	invalid value	invalid value		invalid value	invalid value	invalid value	invalid value
Trainscg	0.758	2.304	-0.127	7.53%		0.6669	2.6771	-0.6256	8.59%
Traingdx	0.7121	2.7276	0.2809	9.20%		0.6352	3.0675	-0.8454	9.95%
Trainbfg	0.7194	2.5736	-0.2263	8.48%		0.5801	3.0321	-1.3856	9.87%
Traincgb	0.7894	1.9795	-0.0242	6.27%		0.7593	1.9396	-0.6985	5.98%

Number of Hidden Layer Neurons:8	Training Set					Validation Set			
	R2	MAE	MBE	MAPE		R2	MAE	MBE	MAPE
Levenberg-Marquardt (trainlm)	0.7931	1.7957	-0.8311	5.66%		0.7778	2.4665	-0.9444	7.85%
Traingd	0.1638	4.2336	0.6864	14.24%		0.3871	4.6347	0.7688	15.80%
Trainscg	0.7933	1.8585	-0.124	5.85%		0.8159	2.2656	-0.3817	7.44%
Traingdx	0.5902	3.0129	0.1025	9.86%		0.5896	3.2927	-0.4136	10.64%
Trainbfg	0.7958	1.6131	0.0484	5.01%		0.8489	1.8743	0.0669	6.17%
Traincgb	0.7346	2.1979	-0.2681	7.05%		0.6799	2.7606	-0.5451	8.95%

Number of Hidden Layer Neurons:9	Training Set					Validation Set			
	R2	MAE	MBE	MAPE		R2	MAE	MBE	MAPE
Levenberg-Marquardt (trainlm)	0.806	2.2456	0.8954	7.46%		0.7479	2.4102	0.9707	7.95%
Traingd	0.4885	3.54	0.2098	11.72%		0.4598	3.4239	0.2843	11.25%
Trainscg	0.8364	1.8162	0.1742	5.84%		0.7748	1.9337	-0.0528	6.07%
Traingdx	invalid value	invalid value	invalid value	invalid value		invalid value	invalid value	invalid value	invalid value
Trainbfg	0.7306	2.4523	-0.0317	8.18%		0.7398	2.3543	-0.0305	7.46%
Traincgb	0.7625	2.3725	0.2742	7.84%		0.6815	2.607	-0.1985	8.05%

Number of Hidden Layer Neurons:10	Training Set					Validation Set			
	R2	MAE	MBE	MAPE		R2	MAE	MBE	MAPE
Levenberg-Marquardt (trainlm)	0.7886	1.9817	-0.0451	6.33%		0.8358	2.2791	-0.4635	7.31%
Traingd	0.2911	4.0293	-0.1929	13.37%		0.3793	4.3876	-0.8296	14.39%
Trainscg	0.6742	2.5439	0.0505	8.38%		0.7877	2.3597	-0.4322	8.22%
Traingdx	0.6078	2.9703	0.1352	9.53%		0.7756	2.5832	0.1093	8.80%
Trainbfg	0.6553	2.8718	-0.3461	9.41%		0.5978	3.6287	-1.1964	11.98%
Traincgb	0.7776	1.7611	-0.2669	5.45%		0.9344	1.4147	-0.3586	4.63%

Number of Hidden Layer Neurons:11	Training Set					Validation Set			
	R2	MAE	MBE	MAPE		R2	MAE	MBE	MAPE
Levenberg-Marquardt (trainlm)	0.853	1.6373	0.2533	5.11%		0.8027	1.9365	0.7668	6.99%
Traingd	0.2933	4.4673	0.2136	14.49%		0.5988	3.066	0.5686	11.28%
Trainscg	0.725	2.4594	-0.22	7.87%		0.5667	2.5136	-0.9169	9.41%
Traingdx	0.7968	2.1154	-0.092	6.68%		0.7638	2.1223	-0.2685	7.64%
Trainbfg	0.7935	2.1077	-0.1208	6.75%		0.7641	2.2041	-0.0812	7.89%
Traincgb	0.8371	1.6363	-0.016	4.95%		0.8523	1.5334	0.2238	5.54%

Number of Hidden Layer Neurons:12	Training Set					Validation Set			
	R2	MAE	MBE	MAPE		R2	MAE	MBE	MAPE
Levenberg-Marquardt (trainlm)	0.721	2.4074	-1.0334	7.66%		0.7275	2.1595	-0.9745	6.53%
Traingd	0.2634	4.5163	0.1494	15.06%		0.1423	4.9127	0.198	16.24%
Trainscg	0.8382	1.6081	-0.0043	5.20%		0.8142	1.7187	0.0343	5.33%
Traingdx	invalid value	invalid value	invalid value	invalid value		invalid value	invalid value	invalid value	invalid value
Trainbfg	0.8139	1.9116	0.1681	6.22%		0.8416	1.897	-0.0506	5.93%
Traincgb	0.8225	1.6805	0.0101	5.44%		0.8254	1.8448	-0.0217	5.76%

Number of Hidden Layer Neurons:13	Training Set					Validation Set			
	R2	MAE	MBE	MAPE		R2	MAE	MBE	MAPE
Levenberg-Marquardt (trainlm)	0.7739	2.116	0.9792	7.15%		0.8093	2.3033	1.094	7.77%
Traingd	invalid value	invalid value	invalid value	invalid value		invalid value	invalid value	invalid value	invalid value

Trainscg	0.7764	2.013	-0.2729	6.35%		0.8486	2.0993	0.0799	7.00%
Traingdx	invalid value	invalid value	invalid value	invalid value		invalid value	invalid value	invalid value	invalid value
Trainbfg	0.7979	1.6521	-0.3765	5.04%		0.8732	1.8115	-0.0612	5.84%
Traincgb	0.809	1.7639	0.033	5.57%		0.8761	1.7814	0.4217	5.86%

Number of Hidden Layer Neurons:14	Training Set					Validation Set			
	R2	MAE	MBE	MAPE		R2	MAE	MBE	MAPE
Levenberg-Marquardt (trainlm)	0.8442	1.589	-0.1955	5.11%		0.8003	1.9845	-0.5522	6.25%
Traingd	0.5434	3.4158	-0.1206	11.44%		0.3645	3.9021	-0.1831	12.97%
Trainscg	0.7682	2.2659	-0.1707	7.26%		0.7002	2.7074	-0.7198	8.51%
Traingdx	0.6559	2.885	0.0685	9.59%		0.6766	2.9539	0.3041	9.82%
Trainbfg	0.8149	1.765	-0.1824	5.56%		0.8152	2.0036	-0.5564	6.26%
Traincgb	0.7727	2.088	0.0719	6.68%		0.7241	2.7173	-0.2916	8.86%

Number of Hidden Layer Neurons:15	Training Set					Validation Set			
	R2	MAE	MBE	MAPE		R2	MAE	MBE	MAPE
Levenberg-Marquardt (trainlm)	0.8231	1.8166	-0.6325	5.65%		0.7884	1.8908	-0.1562	6.30%
Traingd	0.2161	4.5379	0.249	14.93%		0.2186	3.9232	0.6078	13.10%
Trainscg	0.7729	2.33	0.0399	7.54%		0.6423	2.3294	0.6865	7.43%
Traingdx	0.4475	3.6799	-0.2989	12.46%		0.3532	3.2803	0.6024	11.61%
Trainbfg	0.6048	3.2408	-0.0945	10.64%		0.4969	3.5224	1.3701	12.62%
Traincgb	0.731	2.6063	0.1148	8.47%		0.738	2.5863	1.1215	9.21%
